# Synthesis of diN-Substituted Glycyl-Phenylalanine Derivatives by Using Ugi Four Component Reaction and Their Potential as Acetylcholinesterase Inhibitors

**DOI:** 10.3390/molecules24010189

**Published:** 2019-01-06

**Authors:** Luis Prent-Peñaloza, Alexander F. de la Torre, José L. Velázquez-Libera, Margarita Gutiérrez, Julio Caballero

**Affiliations:** 1 Organic Synthesis Laboratory and Biological Activity (LSO-Act-Bio), Institute of Chemistry of Natural Resources, Universidad de Talca, Casilla 747, Talca 3460000, Chile; luisprent@gmail.com (L.P.-P.); mgutierrez@utalca.cl (M.G.); 2 Departamento de Química Orgánica, Facultad de Ciencias Químicas, Universidad de Concepción, Concepción 4030000, Chile; afndz1982@gmail.com; 3 Centro de Bioinformática y Simulación Molecular (CBSM), Universidad de Talca, Casilla 747, Talca 3460000, Chile; josevlibera2010@gmail.com

**Keywords:** acetylcholinesterase inhibitors, molecular docking, multicomponent reactions, N-substituted phenylalanine derivatives, Ugi-4CR

## Abstract

Ugi four component reaction (Ugi-4CR) isocyanide-based multicomponent reactions were used to synthesize diN-substituted glycyl-phenylalanine (diNsGF) derivatives. All of the synthesized compounds were characterized by spectroscopic and spectrometric techniques. In order to evaluate potential biological applications, the synthesized compounds were tested in computational models that predict the bioactivity of organic molecules by using only bi-dimensional molecular information. The diNsGF derivatives were predicted as cholinesterase inhibitors. Experimentally, all of the synthesized diNsGF derivatives showed moderate inhibitory activities against acetylcholinesterase (AChE) and poor activities against butyrylcholinesterase (BuChE). Compound **7a** has significant activity and selectivity against AChE, which reveals that the diNsGF scaffold could be improved to reach novel candidates by combining other chemical components of the Ugi-4CR in a high-throughput combinatorial screening experiment. Molecular docking experiments of diNsGF derivatives inside AChE suggest that these compounds placed the phenylalanine group at the peripheral site of AChE. The orientations and chemical interactions of diNsGF derivatives were analyzed, and the changeable groups were identified for future exploration of novel candidates that could lead to the improvement of diNsGF derivative inhibitory activities.

## 1. Introduction

Multicomponent reactions (MCRs) have clear advantages with respect to conventional multistep reactions due to their potential to synthesize complicated organic molecules with low costs and quick reaction times [1,2]. It is common to use MCRs to obtain biologically relevant scaffold structures, mainly isocyanide-based MCRs, where the Ugi four component reaction (Ugi-4CR) has been particularly important for these endeavors [3,4,5,6,7,8]. Ugi-4CRs are easy and straightforward to perform; isocyanides derived from enantiopure α-amino acids (isocyanoacetates) could serve as substrates for this reaction. There are many successful reports that have obtained relevant biological scaffolds by using isocyanoacetate-based Ugi-4CRs [9,10].

The use of Ugi-4CRs by using isocyanoacetates as substrates has been documented in some diverse examples in the last few decades. For instance, Bonne et al. reported the development of a three-component synthesis of 5-methoxyoxazoles and a four-component synthesis of furopyrrolones on the basis of the unique reactivity of methyl α-(*p*-nitrophenyl)-α-isocyanoacetate [11]. On the other hand, Nenajdenko et al. reported the use of a 4-methyl-2,6,7-trioxabicyclo(2.2.2)octyl derivative (OBO-ester) of isocyanoacetic acid instead of an ethyl ester of R-isocyanoacids compounds in Ugi-4CR with subsequent deprotection that gives access to dipeptides with preserved configuration at the C-terminal amino acid [12]. The same authors synthesized fully protected natural and unnatural N-acetylcysteine, dipeptide Cys–Gly, glutathione, and homoglutathione derivatives by the Ugi-4CR using various benzylthio aldehydes and ketones as carbonyl building blocks. They used the ethyl isocyanoacetate as a peptide synthesis precursor, but they replaced it by the OBO-ester of the isocyanoacetic acid in cases where higher reactivity of an isocyanide was required [13]. In another report, Zhou et al. developed a protocol in which cyclohexylisocyanide and methyl 2-isocyanoacetate were used as convertible isocyanides for the Ugi/de-Boc/cyclization/Suzuki synthesis of biaryl-substituted 1,4-benzodiazepine-2,5-diones [14]. Finally, it is relevant to mention the approach described by Kotha [15] involving solid–liquid phase-transfer-catalyzed dialkylation of ethyl isocyanoacetate to synthesize symmetrically α,α-disubstituted α-amino acids.

The use of isocyanoacetates as Ugi-4CR substrates is relevant at present for the synthesis of novel bioactive compounds. In this work, diN-substituted glycyl-phenylalanine (diNsGF) derivatives were synthesized by using isocyanoacetate-based Ugi-4CRs. To identify possible biological applications of the novel compounds, they were tested in computational predictive models previously developed by our group. In these models, their possible role as cholinesterase inhibitors was detected. Our compounds were evaluated against the enzymes acetylcholinesterase (AChE) and butyrylcholinesterase (BuChE). Experimental biological activities and molecular modeling experiments demonstrate that the diNsGF derivatives have potential for the design of future potent AChE inhibitors, where the presence of the phenylalanine group guarantees the selectivity of these compounds for AChE versus BuChE.

## 2. Results

### 2.1. Synthesis of diN-Substituted Glycyl-Phenylalanine (diNsGF) Derivatives

**Scheme 1:**
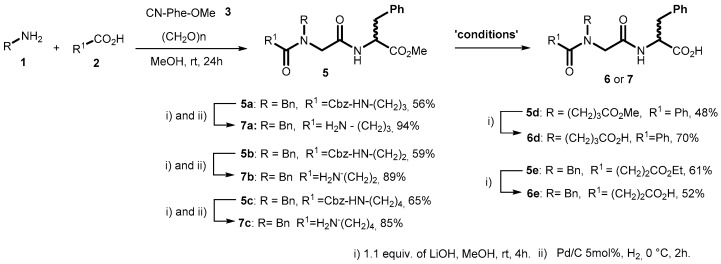
The Ugi four component reaction (Ugi-4CR) for the synthesis of diNsGF derivatives.

An isocyanide MCR approach based on the well-known Ugi-4CR protocol [16] was implemented for the development of new diNsGF derivatives. Two different elements of diversity were varied for the preparation of these compounds, i.e., the carboxylic acid and the amino components, while the DL-Phenylalanine ethyl ester isocyanide and paraformaldehyde were unchanged as shown in Scheme 1. Five novel diNsGF derivatives were synthesized in moderate to good yields by a two-step procedure, followed by either N-terminus or C-terminus deprotection (Scheme 1). The pure compounds were obtained after flash chromatographic purification and their purity was confirmed by spectroscopy characterization.

In an initial approach, a solution-phase Ugi-4CR protocol was employed for the synthesis of diNsGF derivatives, which proved to be suitable for β, γ, and δ amino acids (compounds **7a**–**c**) but gave poor results when all reagents were added in one step. To solve this problem, we preformed the imine for 1 h and then the acid and isocyanide component was added, which provided good efficiency in the multicomponent preparation of the previously designed diNsGF compounds as compared with the literature [17]. Then, Cbz-protected compounds **7a**–**c** were selected for the ester deprotection, employing a basic hydrolysis with LiOH in methanolic solution. Followed by catalytic hydrogenation using palladium in carbon, the zwitterion pseudo-peptide amino acids **7a**–**c** were succesfully obtained. By using the previously described methodology, succinic acid monomethyl ester and benzoic acid were employed to the synthesis of compounds 6d and 6e, respectively. It was possible to obtain two novel dicarboxylic acid derivatives in good overall yield after both ester protected groups in the structure skeleton were deprotected, employing a basic hydrolysis with LiOH in methanolic solution (Scheme 1).

The novel diNsGF derivatives were fully characterized by spectroscopy (FT-IR, ^1^H, ^13^C-NMR, and two-dimensional NMR) and mass spectrometry. The FT-IR spectra of **7a**–**c** and **6d**–**e** were compared to each other, clearly showing the incorporation of the peptidic moiety for all Ugi adducts (see the Supplementary Material). For example, the new bands appearing at 2930 cm^−1^ and 1700 cm^−1^ correspond to the Csp^3^-H and the carbonyl stretching, respectively. Similarly, there are other new bands, such as the amine stretching and the band around 1420 cm^−1^, which were also attributed to aliphatic segments for **7a**–**c**.

The NMR spectra were similar for all compounds. The signal of chiral carbon that appears at 56 ppm from the Attached Proton Test (APT) experiment was the key to the elucidation of the structure, especially in the Heteronuclear Single Quantum Coherence (HSQC) experiment. This signal allowed us to begin the assigment of carbons and protons, and, therefore, to define the structures with the help of another experiment (mono and two-dimensional). The mass of each compound was obtained with a deviation less than 3 ppm, and spectra did not show any fragmentation. This illustrates the stability of this class of compounds against the strong conditions in which these experiments were performed (see the Supplementary Material Figures S1–S40).

### 2.2. Predictions of the AchE Inhibitory Activities by Using Two-Dimensional (2D) Autocorrelation Models

The idea was to develop novel compounds with a potential biological activity designed by two possible approaches. Firstly, compounds were designed with both amino and carboxylic groups to improve the interactions with the target by acting like a zwitterion pseudo-peptide amino acid (compounds **7a**–**c**). Secondly, similar compounds were designed with two carboxylic groups (compounds **6d** and **6e**). Following our protocol, we planned to identify potential activities for the synthesized compounds.

With this in mind, we used the 2D autocorrelation models recently reported in reference [18] to predict the potential AChE inhibitory activities of the synthesized diNsGF derivatives. These models were trained with diverse AChE inhibitors with activities between pIC_50_ 2 and 10, where IC_50_ was measured in M. According to these models, compounds **7a**–**c** and **6d**–**e** were predicted to have activities between pIC_50_ 6 and 7.5, which indicates that they could inhibit the AChE enzyme.

A question arose about the capacities of our 2D autocorrelation models to predict diNsGF derivatives. For this, we tested whether the applicability domain (AD) of these models included the novel synthesized structures. The AD was previously estimated for the 2D autocorrelation models to evaluate their ability to tolerate new molecules by using William’s plots [19]. We could confirm that the diNsGF derivatives were inside the AD if the leverages of their independent 2D autocorrelation variables were within the range of the leverages of the molecules used for training the models. It means that calculated leverages should be below the warning leverage h*, which had values around 0.34 and 0.38 for the previously developed models.

The calculated leverages for compounds **7a**–**c** and **6d**–**e** were below 0.34 for the tested models. An exception was found only for compound **6e**, which had a leverage value of 0.44 (higher than the warning leverage h*) for one of the 2D autocorrelation models. However, it was included within the AD range of the remaining models.

After these computational experiments, we concluded that our 2D autocorrelation models predicted that the diNsGF derivatives are potential AChE inhibitors. We consider these predictions to be reliable since the studied compounds are included in the AD of the predictive models.

### 2.3. Results of the AChE and BuChE Inhibitory Activities

The previously synthesized compounds were tested against AChE and BuChE. As shown in Table 1, the new diNsGF compounds had AChE inhibitory activities in a micromolar range. AChE inhibitory potency was expressed as the half of maximal inhibitory concentration, IC_50_. The inhibitory activity was determined by the modified Ellman’s method. The alkaloid galantamine was used as the reference drug. Table 1 shows that most of the studied compounds were able to inhibit in vitro both enzymes AChE and BuChE but in a very broad range of concentrations. At the same time, it proves that all new synthesized diNsGF compounds are significantly more active towards AChE than BuChE, with the exception of compound **6e**. This result confirms that the previously reported 2D autocorrelation models had the capacity to identify novel active compounds against AChE. With respect to the moderate activities found for the synthesized compounds, we consider that the newly synthesized diNsGF derivatives can serve as base molecules for the design of novel selective AChE inhibitors.

Compounds **6d**, **7a**, **7b**, and **7c** showed good AChE inhibitory activities, with IC_50_ values of 10.64, 3.67, 41.31, and 12.56 µM, respectively. Meanwhile, compound **6e** showed the lowest IC_50_ value against BuChE (135 µM). Compounds **7a**, **7b**, and **7c** have free amine groups attached to carbon chains of three, two, and four methylene groups, respectively. The differences in IC_50_ values for these compounds reflect possible effects of the chain length for improving the interactions that their amino groups may have with residues of the AChE binding site. The distance achieved by the amino group through three methylene groups (compound **7a**) was adequate to achieve stable interactions between the inhibitor and the enzyme, enhancing its inhibitory capacity and its selectivity for AChE.

An in silico analysis (ADME/Tox) was done to predict some properties of the novel diNsGF derivatives (Table 1). The properties that have values within the acceptable ranges are: (i) logP_o/w_, which estimates how the behavior of each molecule will be in terms of lipophilicity [20] and permeability though the membrane [21]; (ii) logS, which indicates the drug transport, uptake, and bioavailability [22]; (iii) human oral absorption, and (iv) logKp (skin permeability), which indicates possibilities of administration and effects on bioavailability [23], a risk assessment of hazardous chemicals, and measurements of transdermal drug release rates [24,25]. The relevance of in silico ADME/Tox assays is that these theoretical features help to delimit the space within which there is a greater probability of finding compounds with a tendency to achieve a successful clinical development [26].

### 2.4. Results of the Kinetic Assays

**Figure 1:**
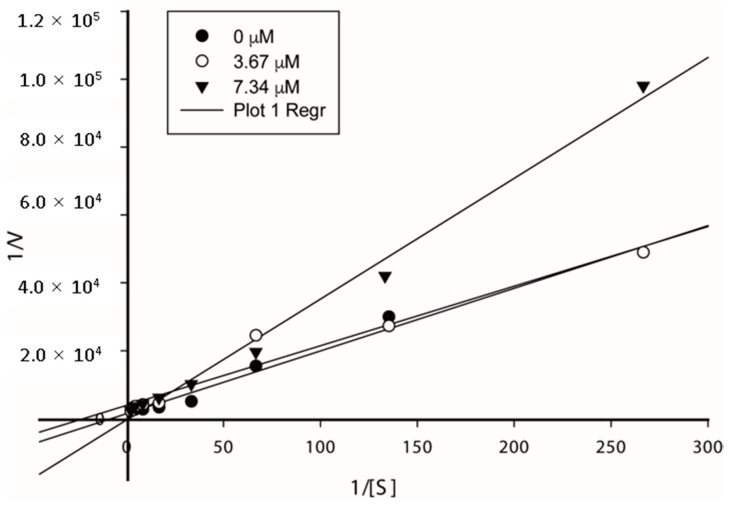
The Lineweaver–Burk plot of AChE (0.02 U) with an acetylthiocholine substrate in the absence and presence of inhibitor **7a**.

Based on the obtained results in the cholinesterase inhibition, and with the aim to assess the kinetic mode of AChE inhibition of the target compounds, the most active compound **7a** was subjected to a kinetic study. For this purpose, the rate of enzyme activity was measured at eight different concentrations of substrate ATC. The obtained Michaelis–Menten parameters along with the Lineweaver–Burk plots (Figure 1) allowed us to conclude that compound **7a** would display a mixed-type inhibition against AChE with variation in Km and Vmax values. The compound **7a** showed Km values of 0.045, 0.125, and 1.049 μM and Vmax of 2.95 × 10^−3^, 2.56 × 10^−4^, and 6.72 × 10^−4^ mol/min, respectively.

### 2.5. Docking Results

The synthesized diNsGF derivatives were predicted as AChE inhibitors by using 2D autocorrelation models, and this result was experimentally confirmed. Their activities were in the micromolar range; therefore, the synthesized compounds could be classified as moderate inhibitors. After the identification of the diNsGF derivatives as AChE inhibitors, the next step was the rational modification of substituents to improve the activities. With this in mind, it had become pertinent to study the putative interactions between our synthesized compounds and the binding site of AChE.

**Figure 2:**
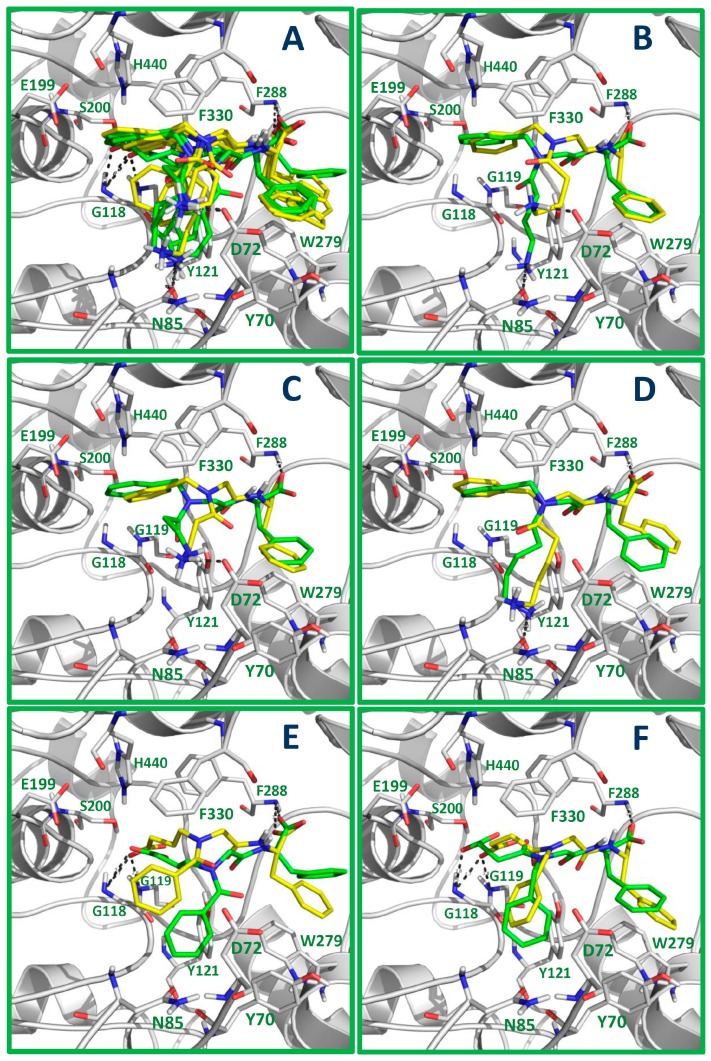
Molecular docking models for diNsGF derivatives inside the AChE binding site. Docking poses of all the compounds (**A**); Docking poses of compounds **7a** (**B**), **7b** (**C**), **7c** (**D**), **6d** (**E**), and **6e** (**F**), where R and S enantiomers are represented in green and yellow, respectively. Protein residues that form the main interactions with the inhibitors are in stick representation.

Therefore, the synthesized diNsGF derivatives (R and S enantiomers) were docked inside the AChE binding site to understand their ligand–protein interactions at the atomic level. The docking results are depicted in Figure 2. As can be seen in Figure 2A, all compounds (considering one or both enantiomers) placed the phenylalanine group at the peripheral site of the AChE pocket, forming a hydrogen bond (HB) between the carboxylate of the ligands and the backbone NH group of the residue F288, and π–π stacking interactions between the phenyl group of the ligands and the indole of the residue W279. This π–π stacking interaction with the residue W279 has been previously described for the AChE–donepezil complex [27] and other AChE inhibitors reported in the literature [28,29]. As in other AChE inhibitors, this interaction explains why the majority of diNsGF derivatives exhibited selectivity for AChE over BuChE. In BuChE, the peripheral site contains alanine instead of tryptophane (A277 instead of W279); therefore, the π–π stacking interaction between diNsGF derivatives and the peripheral site of AChE cannot be established between these compounds and BuChE.

The compounds that contain an NH_3_^+^ group (**7a, 7b**, and **7c**) can form HBs with the side chain CO of the residue N85 (Figure 2B,D) or a salt bridge with the side chain carboxylate of D72 (Figure 2B,C). At the same time, these compounds form a cation–π interaction between the phenyl of the benzylamine of the ligand and the positively charged imidazole of the residue H440 (Figure 2B–D). An HB interaction was formed with the side chain CO of the residue N85 when the NH_3_^+^ group was a large linear chain containing four methylene groups (compound **3**, Figure 2D), while a salt bridge was formed with D72 when the NH_3_^+^ group was a short linear chain containing two methylene groups (compound **2**, Figure 2C). When the NH_3_^+^ group was a linear chain containing three methylene groups, both possibilities were obtained (one for each enantiomer) according to docking experiments (compound **1**, Figure 2B). The compounds that contain carboxyalkyl groups at the N of the benzoylamide (**6d**) or benzylamine (**6e**) can form HBs with the side chain CO of the residues G118 and G119 (Figure 2E,F), while the phenyl groups are oriented to the pocket near the residues Y121, D72, and W84.

## 3. Materials and Methods

### 3.1. Preparation of Compounds

The starting material was purchased from either Merck or Sigma-Aldrich. Monitoring of the reactions was through thin layer chromatography (TLC) using silica gel 60 (Merck, Darmstadt, Germany). Melting points (uncorrected) were determined on an Electrothermal IA9100 melting point apparatus (Stone, Staffs, UK). All products were characterized by spectral data (IR, ^1^H and ^13^C NMR, MS). ^1^H and ^13^C NMR spectra (400 MHz for ^1^H and 100 MHz for ^13^C) were recorded on an AM-400 spectrometer (Bruker, Rheinstetten, Germany) using DMSO-*d*_6_ as solvents. The chemical Shift (δ) of the solvent is reported at 2.5 for ^1^H-NMR and 40.09 for ^13^C-NMR, δ and *J* values are reported in ppm and Hz, respectively. The signals are represented as follows: Singlet (s), doublet (d), triplet (t), multiplet (m). IR spectra (KBr pellets, 500–4000 cm^−1^) were obtained from a NEXUS 670 FT-IR spectrometer (Thermo Nicolet, Madison, WI, USA). High-resolution mass spectra (HRMS-ESI) were obtained from Thermo Fisher Scientific Exactive Plus mass spectrometer. The analysis was performed at heater temperature, 50 °C; sheath gas flow, 5; sweep gas flow rate, 0, and spray voltage, 3.0 kV at negative mode. The accurate mass measurements were performed at a resolution of 140,000. The absorbance of biological assay was measured at 405 nm using a microtiter plate reader (Multiskan EX, Thermo, Vantaa, Finland). ADME7Tox prediction was made with QuikProp module of the Schrodinger suite (Schrödinger, LLC: New York, NY, USA).

(*S*)-Ethyl-2-Amino-3-Phenylpropionate: This compound was prepared following the methodology reported by Guo et al. [30]. SOCl_2_ (5.88 mL) was added dropwise at 0 °C to a suspension of L-phenylalanine **1** (4.46 g, 27.0 mmol) in 50 mL ethanol. The resulting mixture was then refluxed for 4 h. The volatiles were concentrated under reduced pressure and the resulting crude was treated with saturated aqueous Na_2_CO_3_ until pH 8–9. The mixture was extracted with Ethyl Acetate (2 × 50 mL). The combined organic phase was dried over Na_2_SO_4_ and evaporated under reduced pressure, obtaining the product as a white solid (5.6 g, 97%).

*N*-Formyl-l-Phenylalanine Ethyl ester: This compound was prepared following the methodology reported by Monge et al. [31]. Acetic anhydride (14 mL, 21 mmol, 7 equiv.) was added dropwise to a solution of (*S*)-Ethyl-2-Amino-3-Phenylpropionate (4.0 g, 21 mmol, 1 equiv.) in Formic acid (39 mL, 21 mmol, 50 equiv.) at 0 °C. The reaction was stirred at room temperature (rt) for 2 h, quenched with H_2_O (40 mL), and then extracted with CH_2_Cl_2_ (5 × 70 mL). The combined organic layers were washed with NaHCO_3_ (sat.) (5 × 70 mL) brine, dried (Na_2_SO_4_), and evaporated under reduced pressure to give the product as a colorless oil (4.4 g, 95%).

Ethyl 2-isocyano-3-phenylpropionate: This compound was prepared following the methodology reported by Monge et al. [31]. Et_3_N (14 mL, 20 mmol, 5 equiv.) was added to a stirred solution of *N*-Formamide ester (4.4 g, 20 mmol, I equiv.) in dry THF (50 mL) at −20 °C under a nitrogen atmosphere. A solution of POCl_3_ (2.3 mL, 20 mmol, 1.3 equiv.) in dry THF (10 mL) was added dropwise and the reaction mixture was allowed to warm to 0 °C. After stirring for 2 h, ice-cold water (65 mL) was added and the mixture was extracted with Et_2_O (4 × 50 mL). The combined organic layers were washed with brine, dried (Na_2_SO_4_), and evaporated under reduced pressure, and the resulting crude product was purified by flash column chromatography on silica gel to give the product as a yellow oil (3.2 g, 79%).

General Ugi-4CR-Based Procedure: A suspension of the amine (0.5 mmol, 1 equiv.) and the aldehyde (0.5 mmol, 1 equiv.) in MeOH (1 mL) was stirred for 1 h at rt. Et_3_N (0.5 mmol, 1 equiv.) was added when α-amino acid methyl ester hydrochlorides were employed. The carboxylic acid (0.5 mmol, 1 equiv.) and the isocyanide (0.5 mmol, 1 equiv.) were then added, and the reaction mixture was stirred at rt for 24 h. The volatiles were concentrated under reduced pressure and the resulting crude product was dissolved in 50 mL of CH_2_Cl_2_. The organic phase was washed sequentially with a saturated aqueous solution of citric acid (25 mL), aqueous 10% NaHCO_3_ (25 mL), and brine (25 mL), and then dried over anhydrous Na_2_SO_4_ and concentrated under reduced pressure. The resulting amorphous solid was used in the deprotection step without further purification [17].

General procedure for ethyl ester deprotection: A solution of lithium hydroxide (0.25 mmol, 2 equiv.) in water (1 mL) was added to a solution of the appropriate ester (0.25 mmol, 1 equiv.) in THF (1 mL). The mixture was homogenized with methanol (1 mL) and stirred at rt for 2 h. The solvent was evaporated to dryness and the residue was dissolved in water. The pH was set acidic with 1 M HCl and the desired product was extracted with EtOAc. The combined organic phases were washed with brine, dried with anhydrous Na_2_SO_4_, and the solvent removed in vacuo. The crudes were purified employing column chromatography. The desired products were confirmed for thin-layer chromatography (TLC) revelated with bromocresol green indicator [32].

General procedure for Cbz deprotection [33]: Pd/C (5 wt%, 5 mol%) was added to a solution of **5a**–**c** (0.96 mmol) in THF:MeOH (1:1, 10 mL) and the mixture was stirred under a hydrogen atmosphere at rt for 1 h. After that, the mixture was filtered through celite (Merck, Darmstadt, Germany), the filtrate was evaporated, and the resultant residue was purified by column chromatography on silica gel (CHCl_3_:MeOH 9:1) to produce compounds **7a**–**c**.

*10-Amino-2,6-dibenzyl-4,7-dioxo-3,6-diazadecanoic acid* (compound **7a**): a suspension of the benzylamine (54.6 µL, 0.5 mmol) and the *p*-formaldehyde (15 mg, 0.5 mmol) in MeOH (1 mL) was stirred for 1 h at rt. After, 4-(((benzyloxy)carbonyl) amino)butanoic acid (118.55 mg, 0.5 mmol) and the ethyl 2-isocyano-3-phenylpropionate (101.5 mg, 0.5 mmol) were then added, and the reaction mixture was stirred at rt for 24 h according to the Ugi-4CR-based procedure. The resulting Cbz and ethyl protected compound was unprotected through the general deprotection procedure. Flash column chromatography purification (DCM:MeOH 5:1) allowed us to obtain **7a** as a mixture of conformers. Yield: 93% (52 mg); white solid; m.p.: 212.5–214.5 °C; IR-FT (KBr, cm^−1^): 3440, 3025, 2921, 2853, 1638, 1625, 1580, 1400; ^1^H NMR (400 MHz, DMSO-*d*_6_) δ 7.86 (d, *J* = 7.6 Hz, 1H), 7.29 (d, *J* = 7.1 Hz, 2H), 7.25–7.12 (m, 8H), 4.61 (d, *J* = 15.2 Hz, 1H), 4.25 (d, *J* = 15.0 Hz, 1H), 4.20 (s, 1H), 3.90 (d, *J* = 17.6 Hz, 1H), 3.70 (d, *J* = 17.4 Hz, 1H), 3.13 (d, *J* = 10.7 Hz, 1H), 2.83 (d, *J* = 10.7 Hz, 1H), 2.79 (s, 3H), 2.51 (s, 2.11H), 1.81 (s, 2H); ^13^C NMR (100 MHz, DMSO) δ 174.80, 172.83, 168.10, 139.99, 138.23, 129.92, 129.62, 129.19, 128.21, 128.14, 127.47, 127.31, 126.26, 56.37, 50.27, 50.20, 38.73, 38.08, 29.81, 23.27. HRMS (ESI, *m*/*z*): calculated for C_22_H_26_N_3_O_4_^−^ [M − H]^−^ 396.1929 found 396.1926.

*9-Amino-2,6-dibenzyl-4,7-dioxo-3,6-diazanonanoic acid* (compound **7b**); a suspension of the benzylamine (54.6 µL, 0.5 mmol) and the *p*-formaldehyde (15 mg, 0.5 mmol) in MeOH (1 mL) was stirred for 1 h at rt. After, 3-(((benzyloxy)carbonyl) amino)propanoic acid (118.6 mg, 0.5 mmol) and the ethyl 2-isocyano-3-phenylpropionate (101.5 mg, 0.5 mmol) were then added, and the reaction mixture was stirred at rt for 24 h according to the Ugi-4CR-based procedure. The resulting Cbz and ethyl protected compound was unprotected through the general deprotection procedure. Flash column chromatography purification (DCM:MeOH 5:1) allowed us to obtain **7b** as a mixture of conformers. Yield: 88% (51 mg); white solid; m.p.: 213.4–215.1 °C; IR-FT (KBr, cm^−1^): 3482, 3054, 3017, 2921, 1653, 1596, 1387; ^1^H NMR (400 MHz, DMSO-*d*_6_) δ 7.88 (d, *J* = 8.08 Hz, 1H_a_), 7.30 (d, *J* = 7.40 Hz, 2H), 7.23–7.17 (m, 8H), 4.55 (d, *J* = 15.11 Hz, 1H), 4.22 (m, 1H), 4.20 (d, *J* = 15.48 Hz, 1H), 3.91 (d, *J* = 17.43 Hz, 1H), 3.77 (d, 1H), 3.12 (d, *J*_1_ = 4.13 Hz, *J*_1_ = 13.62 Hz, 1H), 2.99 (m, 2H), 2.82 (d, *J*_1_ = 8.88 Hz, *J*_1_ = 13.64 Hz, 1H), 2.69 (m, 2H). ^13^C NMR (100 MHz, DMSO) δ 174.45, 171.48, 167.50, 139.81, 137.82, 129.70, 129.19, 128.85, 128.33, 128.25, 127.58, 127.42, 126.26, 56.26, 50.21, 49.79, 38.15, 35.88, 30.89. HRMS (ESI, *m*/*z*): calculated for C_21_H_24_N_3_O_4_^−^ [M − H]^−^, 382.1772 found 382.1771.

*11-Amino-2,6-dibenzyl-4,7-dioxo-3,6-diazaundecanoic acid* (compound **7c**); a suspension of the benzylamine (54.6 µL, 0.5 mmol) and the *p*-formaldehyde (15 mg, 0.5 mmol) in MeOH (1 mL) was stirred for 1 h at rt. After, 5-(((benzyloxy)carbonyl) amino)pentanoic acid (125.6 mg, 0.5 mmol) and the ethyl 2-isocyano-3-phenylpropionate (101.5 mg, 0.5 mmol) were then added, and the reaction mixture was stirred at rt for 24 h according to the Ugi-4CR-based procedure. The resulting Cbz and ethyl protected compound was unprotected through the general deprotection procedure. Flash column chromatography purification (DCM:MeOH 5:1) allowed us to obtain **7c** as a mixture of conformers. Yield: 85% (56 mg); white solid; m.p.: 218.9–220.5 °C; IR-FT (KBr, cm^−1^): 3448, 3062, 3028, 2926, 1659, 1640, 1627, 1387, 1209, 1026; ^1^H NMR (400 MHz, DMSO-*d*_6_) δ 7.75 (d, *J* = 7.93 Hz, 1H), 7.32–7.16 (m 10H), 4.45 (d, *J* = 15.18 Hz, 1H), 4.28 (d, *J* = 15.08 Hz, 1H), 4.21 (d, *J*_1_ = 8.01 Hz, *J*_2_ = 12.81 Hz, 1H), 3.85 (d, *J* = 17.24 Hz, 1H), 3.75 (d, *J* = 17.79 Hz, 1H), 3.09–3.11 (d, 1H), 2.85 (dd, *J*_1_ = 8.15 Hz, *J*_2_ = 13.49 Hz, 1H), 2.72 (m, 2H), 2.26 (m, 2H), 1.55 (m, 4H). ^13^C NMR (101 MHz, DMSO) δ 174.36, 173.14, 167.53, 139.59, 138.18, 129.70, 129.21, 128.85, 128.30, 128.20, 128.12, 127.20, 126.29, 56.02, 50.13, 49.60, 38.70, 38.12, 31.90, 27.22, 21.81. HRMS (ESI, *m*/*z*): calculated for C_23_H_28_N_3_O_4_^−^ [M − H]^−^, 410.2085 found 410.2083.

*6-Benzamido-2-benzyl-4-oxo-3,6-diazadecanedioic acid* (compound **6d**); triethylamine (69.7 µL, 0.5 mmol) was added to a suspension of methyl 4-aminobutanoate (76.8 mg, 0.5 mmol) in MeOH (1 mL), stirring for 10 min. Then, *p*-formaldehyde (15 mg, 0.5 mmol) was added and the mixture was stirred for 1 h at rt. After, benzoic acid (61 mg, 0.5 mmol) and the ethyl 2-isocyano-3-phenylpropionate (101.5 mg, 0.5 mmol) were added, and the reaction mixture was stirred at rt for 24 h according to the Ugi-4CR-based procedure. The resulting ethyl and methyl protected compound was unprotected through the general deprotection procedure. Flash column chromatography purification (DCM:MeOH 3:1) allowed us to obtain compound **6d** as a mixture of conformers. Yield: 70% (61 mg); white solid; m.p.: 144.3–146 °C; IR-FT (KBr, cm^−1^): 3443, 2921, 2850, 1727, 1667, 1638, 1612; ^1^H NMR (400 MHz, DMSO-*d*_6_) δ 12.37 (s, 1H,), 8.34 (d, *J* = 8.18 Hz, 1H), 7.46–7.12 (m, 10H), 4.50 (m, 1H, H), 3.77 (d, *J* = 5.13 Hz, 2H), 3.20 (m, 2H), 3.06 (m, 1H), 2.88 (m, 1H), 2.23 (t, 2H), 1.73 (t, 2H). ^13^C NMR (101 MHz, DMSO) δ 174.56, 173.12, 171.58, 168.58, 137.93, 136.86, 129.70, 129.53, 128.70, 128.62, 127.03, 126.88, 53.88, 51.66, 45.49, 37.02, 31.68, 22.35, HRMS (ESI, *m*/*z*): calculated for C_22_H_23_N_2_O_6_^−^ [M − H]^−^, 411.1562 found 411.1571.

*2,6-Dibenzyl-4,7-dioxo-3,6-diazadecanedioic acid* (compound **6e**); a suspension of the benzylamine (54.6 µL, 0.5 mmol) and the *p*-formaldehyde (15 mg, 0.5 mmol) in MeOH (1 mL) was stirred for 1 h at rt. After, 4-ethoxy-4-oxobutanoic acid (64 µL, 0.5 mmol) and the ethyl 2-isocyano-3-phenylpropionate (101.5 mg, 0.5 mmol) were added, and the reaction mixture was stirred at rt for 24 h according to the Ugi-4CR-based procedure. The resulting ethyl protected compound was unprotected through the general deprotection procedure. Flash column chromatography purification (DCM:MeOH 3:1) allowed us to obtain compound **6e** as a mixture of conformers. Yield: 52% (60 mg); white solid; m.p.: 163.4–164.8 °C; IR-FT (KBr, cm^−1^): 3461, 2923, 2858, 1745, 1724, 1667, 1638; ^1^H NMR (400 MHz, DMSO-*d*_6_) δ 12.42 (s, 1H), 8.37 (d, *J* = 8.31 Hz, 1H), 7.31–7.13 (m, 10H), 4.54 (dd, *J*_1_ = 4.04 Hz, *J*_2_ = 9.00 Hz, 1H), 4.38 (d, *J* = 15.22 Hz, 1H), 4.28 (d, *J* = 15.23 Hz, 1H), 3.90 (d, *J* = 17.51 Hz, 1H), 3.82 (d, 1H), 3.10 (dd, *J*_1_ = 4.61 Hz, *J*_2_ = 13.77 Hz, 1H), 2.87 (dd, *J*_1_ = 4.76 Hz, *J*_2_ = 9.09 Hz, 1H), 2.62 (t, *J* = 6.50 Hz, 1H), 2.47 (t, *J* = 6.53 Hz, 1H), 2.42 (m, 2H). ^13^C NMR (101 MHz, DMSO) δ 174.39, 173.16, 172.52, 168.38, 137.98, 137.94, 129.61, 129.13, 128.82, 128.65, 128.06, 127.48, 127.31, 126.90, 53.83, 49.56, 49.40, 37.24, 29.67, 27.79, HRMS (ESI, *m*/*z*): calculated for C_22_H_23_N_2_O_6_^−^ [M − H]^−^, 411.1562 found 411.1570.

### 3.2. Computational Predictions of Bioactivities

The molecular structures of the diNsGF derivatives (compounds **7a**, **7b**, **7c**, **6d**, and **6e**) were sketched in MarvinSketch (ChemAxon, Budapest, Hungary). Two-dimensional (2D) autocorrelation descriptors with lags between 1 and 8 were calculated for these structures by using the Dragon software v3.0 (DRAGON, Milano Chemometrics, Milano, Italy). Two-dimensional (2D) autocorrelation descriptors capture the interdependence between atomic properties (masses, van der Waals volumes, electronegativities, and polarizabilities) in a molecular graph [34,35]. The activities of the diNsGF derivatives were predicted by using computational 2D autocorrelation models recently reported by our group, trained for the identification of potential AChE inhibitors [18]. These models were trained with a broad dataset of around 300 AChE inhibitors, and they were validated by using different statistical tests. We previously demonstrated the ability of these models to predict diverse AChE inhibitors; therefore, they were used in the current work for predicting the potential AChE inhibitory activities of the synthesized diNsGF derivatives.

### 3.3. AChE and BuChE Assays

AChE hydrolyzes acetylcholine, which mediates transmission at some synapses in the brain, including synapses involved in the acquisition of short-term memory. Therefore, AChE inhibitors lead to the restoration of the levels of acetylcholine, which is needed for patients with a failing memory, such as Alzheimer’s disease (AD) patients. AChE inhibitors, such as donepezil [27], galantamine [36], and rivastigmine [37], have been approved for AD treatment during the last few decades, but the presence of several side effects has been a challenge for the prolonged use of these drugs [38,39,40]. In the clinical treatment of AD, selective AChE inhibitors have shown better therapeutic effects when compared with nonselective inhibitors. Therefore, the development of novel selective AChE inhibitors is an important approach for AD treatment.

Inhibitory activities were evaluated using the methods by [41]. The samples were dissolved in 96-well plates (50 μL) in phosphate buffer (K_2_HPO_4_ 8 mM, NaH_2_PO_4_ 2.3 mM, NaCl 150 mM, and 0.5% Tween 20, pH 7.6). A solution of acetylcholinesterase (AChE)/butyrylcholinesterase (BuChE) (50 μL, 0.20 unit/mL) from *Electrophorus electricus* and equine serum was added to the same buffer, respectively. The assay solutions, except for the substrate, were pre-incubated with the enzymes for 30 min at room temperature (rt). Then, the substrate solution, composed of Na_2_HPO_4_ (40 mM), acetylthiocholine (ATC)/butyrylthiocholine (BTC) (0.24 mM), and 5,5′-dithio-bis(2-nitrobenzoic acid) (0.2 mM, DTNB, Ellman’s reagent), was added. The absorbance of the reaction was measured at 405 nm for 5 min. AChE/BuChE inhibition was determined for each compound. The enzymatic activity was calculated as a percentage and then compared with the control, which was composed of buffer and an enzyme solution. The compounds were tested in dilution intervals ranging from 500 to 0.98 µg/mL. Each assay was run in triplicate and each reaction was repeated three times. The IC_50_ values were determined using a regression analysis. The alkaloid galantamine was used as the reference compound. The galantamine IC_50_ was developed in three courses ranging from 250 to 0.0.031 μg/mL with an average of 0.57 μM for AchE and 7.96 μM for BuChE. DMSO was diluted to a concentration in excess of 1 in 10,000, and no inhibitory action in neither AChE nor BuChE was detected in separate prior experiments.

### 3.4. Kinetic Assays

The kinetic studies of the most active compound were performed according to Ellman’s method with some modifications to the 96-well plate. The assays were carried out in a total volume of 200 μL per well containing AChE from *Electrophorus electricus* (0.20 unit/mL) in PBS, the synthesized compound in DMSO, PBS at IC_50_, 2× IC_50_ concentration, and substrate solution. The general procedure was as follows: AChE and the synthesized compounds **7a** to IC_50_ and twice concentrations in PBS were mixed and incubated at rt for 30 min. After incubation, substrate from 3.8 × 10^−3^–0.48 mM was added into the mixture. The reaction was recorded for 10 min measuring the absorbance at 405 nm using a Microtiter plate reader (Multiskan EX, Thermo). Data were plotted using GraphPad Prism v4.03 software (GraphPad Software Inc., San Diego, CA, USA) to obtain the Michaelis–Menten constant (Km) and maximal velocity (Vmax) values. Km values were expressed in μM and Vmax as mol/min/mg protein and correspond to the mean ± SD of three independent assays in triplicate.

### 3.5. Molecular Docking

Here, we report the docking experiments that were performed to understand how diNsGF derivatives interact with AChE, which is a necessary task for future rational design for improving AChE inhibitory activities.

Docking was used to obtain the putative binding modes of the diNsGF derivatives inside the AChE binding site. Glide SP and XP methods, contained in Maestro software v9.0 (Schrödinger, LLC: New York, NY, USA), were employed to perform the docking experiments [42,43]. Glide docking searches ligand orientations by using hierarchical filters in a defined receptor grid space. Filters include a sampling of the positional, conformational, and orientational space of the ligands with a final evaluation of their energy interactions with the protein residues inside the grid.

The protein structural coordinates were extracted from the X-ray crystal structure of the AChE–donepezil complex (Protein Data Bank (PDB) code 1EVE). This structure was modified with the Protein Preparation Wizard in Maestro (Schrödinger, LLC: New York, NY, USA): hydrogen atoms were added, followed by the adjustment of bond orders, protonation states for protonatable residues were adjusted to match pH = 7.4, and water molecules were deleted. Finally, it was subjected to geometry optimization by using an OPLS2005 force field [44].

The sketched structures of diNsGF derivatives were converted into three-dimensional structures with the Maestro software facilities. A grid box of 18 Å × 18 Å × 18 Å was centered on the center of mass of the donepezil in the modified protein structure. Ligand minimizations in the receptor field were carried out with a distance-dependent dielectric of 2.0. Afterwards, the lowest energy poses were subjected to a Monte Carlo procedure that sampled the nearby torsional minima, and the best poses were determined by using the GlideScore scoring function, where terms for buried polar groups and steric clashes were considered [45].

## 4. Conclusions

In this work, we report the synthesis of novel diNsGF derivatives through Ugi-4CR in mild conditions, a simple starting material, and moderate yields. The synthesized compounds **7a**–**7c** and **6d**–**6e** were purified and characterized. Their potential use as bioactive compounds was explored by using predictive models. Previously constructed 2D autocorrelation models identified possible AChE inhibitory activities in our compounds; therefore, experiments were prepared to confirm this hypothesis.

The obtained compounds were tested against AChE and BuChE enzymes, showing moderate inhibition values and good selectivity. The binding modes of the compounds were studied by using molecular docking and the essential interactions between the compounds and the AChE binding site were characterized. These models suggest that rational modifications of the substituents at the *N* of the glycine in the diNsGF derivatives (R and R1 groups) based on the described AChE-ligand models provide a basis for the development of novel AChE inhibitors through the use of the Ugi-4CR in a few steps. This facilitates the possibility of exploring many different substituents to improve the interactions between the diNsGF derivatives and the AChE binding site. For the design of novel diNsGF derivatives in the future, researchers could benefit from the main advantages of Ugi-4CRs, such as quick reaction times, diminished costs, and the prevention of time-consuming product purification.

Other dipeptidic derivatives could be explored in the future with an aromatic amino acid instead of the phenylalanine to preserve the π–π stacking interactions with the residue W279 (an interaction that was previously described for the AChE–donepezil complex). The potential of the diNsGF derivatives as AChE inhibitors was demonstrated in this work. Their orientations inside the AChE binding site indicate that the aromatic group present in the phenylalanine is important for the AChE inhibitory activity. In the future, we could explore the use of different aromatic groups in the design of diN-substituted glycyl-tyrosine and glycyl-tryptophan derivatives as AChE inhibitors.

## Figures and Tables

**Table 1:** Inhibitory activity of diNsGF derivatives from AChE and BuChE enzymes. ^1^

Entry	IC_50_ ± SEM (µM) AChE	IC_50_ (µM) BuChE	Selectivity	QPlogPo/w ^2^	QPlogS ^2^	QPlogKp ^2^	Human Oral Absorption ^2^
**7a**	3.67 ± 0.8	243.84 ± 4.6	66.44	−0.527	−2.127	−5.078	2
**7b**	41.31 ± 2.3	461.49 ± 6.2	11.17	0.235	−2.639	−4.401	2
**7c**	12.56 ± 1.6	636.72 ± 3.7	50.69	−0.104	−2.775	−5.071	2
**6d**	10.64 ± 1.5	301.77 ± 5.8	28.36	2.873	−3.249	−3.541	1
**6e**	304.87 ± 5.7	135.85 ± 2.9	0.45	2.246	−2.072	−2.696	2
Galantamine	0.57 ± 0.1	7.96 ± 0.8	13.96				

**^1^** Data are means ± standard error of the mean (SEM) of triplicate independent experiments; ^2^ In silico predicted properties: log P_o/w_ range: (−2)–(6.5), log S range: (−6.5)–(0.5), log Kp range: (−8.0)–(−1.09), human oral absorption: 1 = low, 2 = medium, 3 = high.

## Supplementary Materials

The following are available online. Figure S1: FT-IR spectrum in KBr for **7a**. Figure S2: 400 MHz ^1^H-NMR spectrum in DMSO-*d*_6_ for **7a**. Figure S3: 400 MHz ^13^C-NMR spectrum in DMSO-*d*_6_ for **7a**. Figure S4: 400 MHz APT spectrum in DMSO-*d*_6_ for **7a**. Figure S5: 400 MHz HSQC spectrum in DMSO-*d*_6_ for **7a**. Figure S6: 400 MHz HMBC spectrum in DMSO-*d*_6_ for **7a**. Figure S7: 400 MHz COSY spectrum in DMSO-*d*_6_ for **7a**. Figure S8: High-resolution mass spectrometry-ESI spectrum of **7a**. Figure S9: FT-IR spectrum in KBr for **7b**. Figure S10: 400 MHz ^1^H-NMR spectrum in DMSO-*d*_6_ for **7b**. Figure S11: 400 MHz ^13^C-NMR spectrum in DMSO-*d*_6_ for **7b**. Figure S12: 400 MHz APT spectrum in DMSO-*d*_6_ for **7b**. Figure S13: 400 MHz HSQC spectrum in DMSO-*d*_6_ for **7b**. Figure S14: 400 MHz HMQC spectrum in DMSO-*d*_6_ for **7b**. Figure S15: 400 MHz COSY spectrum in DMSO-*d*_6_ for **7b**. Figure S16: High-resolution mass spectrometry-ESI spectrum of **7b**. Figure S17: FT-IR spectrum in KBr for **7c**. Figure S18: 400 MHz ^1^H-NMR spectrum in DMSO-*d*_6_ for **7c**. Figure S19: 400 MHz ^13^C-NMR spectrum in DMSO-*d*_6_ for **7c**. Figure S20: 400 MHz APT spectrum in DMSO-*d*_6_ for **7c**. Figure S21: 400 MHz HSQC spectrum in DMSO-*d*_6_ for **7c**. Figure S22: 400 MHz HMBC spectrum in DMSO-*d*_6_ for **7c**. Figure S23: 400 MHz COSY spectrum in DMSO-*d*_6_ for **7c**. Figure S24: High-resolution mass spectrometry-ESI spectrum of **7c**. Figure S25: FT-IR spectrum in KBr for **6d**. Figure S26: 400 MHz ^1^H-NMR spectrum in DMSO-*d*_6_ for **6d**. Figure S27: 400 MHz ^13^C-NMR spectrum in DMSO-*d*_6_ for **6d**. Figure S28: 400 MHz APT spectrum in DMSO-*d*_6_ for **6d**. Figure S29: 400 MHz HSQC spectrum in DMSO-*d*_6_ for **6d**. Figure S30: 400 MHz HMQC spectrum in DMSO-*d*_6_ for **6d**. Figure S31: 400 MHz COSY spectrum in DMSO-*d*_6_ for **6d**. Figure S32: High-resolution mass spectrometry-ESI spectrum of **6d**. Figure S33: FT-IR spectrum in KBr for **6e**. Figure S34: 400 MHz ^1^H-NMR spectrum in DMSO-*d*_6_ for **6e**. Figure S35: 400 MHz ^13^C-NMR spectrum in DMSO-*d*_6_ for **6e**. Figure S36: 400 MHz APT spectrum in d_6_-DMSO for **6e**. Figure S37: 400 MHz, HSQC spectrum in DMSO-*d*_6_ for **6e**. Figure S38: 400 MHz, HMQC spectrum in DMSO-*d*_6_ for **6e**. Figure S39: 400 MHz, COSY spectrum in DMSO-*d*_6_ for **6e**. Figure S40: High-resolution mass spectrometry-ESI spectrum of **6e**.
