# Supplementary material for: Synthesis of diN-Substituted Glycyl-Phenylalanine Derivatives by Using Ugi Four Component Reaction and Their Potential as Acetylcholinesterase Inhibitors
Source: Molecules. 2019 Jan 6;24(1):189. doi: 10.3390/molecules24010189 (PMC6337627; doi:10.3390/molecules24010189)
Supplement: Supplementary file 1 [file molecules-24-00189-s001.pdf]

Supplementary Material

Synthesis of diN-Substituted Glycyl-Phenylalanine Derivatives by Using Ugi Four Component Reaction and their Potential as Acetylcholinesterase Inhibitors

Luis Prent-Peñaloza [1], Alexander F. de la Torre [2], José L. Velázquez-Libera [3], Margarita Gutiérrez [1] and Julio Caballero [3,*]

1 Organic Synthesis Laboratory and Biological Activity (LSO-Act-Bio), Institute of Chemistry of Natural Resources, Universidad de Talca, Casilla 747, Talca 3460000, Chile; luisprent@gmail.com (L.P.-P.); mgutierrez@utalca.cl (M.G.)

2 Departamento de Química Orgánica, Facultad de Ciencias Químicas, Universidad de Concepción, Concepción 4030000, Chile; afndz1982@gmail.com

3 Centro de Bioinformática y Simulación Molecular (CBSM), Universidad de Talca, Casilla 747, Talca 3460000, Chile; josevlibera2010@gmail.com

* Correspondence: jcaballero@utalca.cl; Tel.: +56-712-418-850

Table of contents

Figure S1. FT-IR spectrum in KBr for **7a**. 3
Figure S2. 400 MHz ^{1}H-NMR spectrum in d_6-DMSO for **7a**. 3
Figure S3. 400 MHz ^{13}C-NMR spectrum in d_6-DMSO for **7a**. 4
Figure S4. 400 MHz APT spectrum in d_6-DMSO for **7a**. 4
Figure S5. 400 MHz HSQC spectrum in d_6-DMSO for **7a**. 5
Figure S6. 400 MHz HMBC spectrum in d_6-DMSO for **7a**. 5
Figure S7. 400 MHz COSY spectrum in d_6-DMSO for **7a**. 6
Figure S8. High-resolution mass spectrometry -ESI spectrum of **7a**. 6
Figure S9. FT-IR spectrum in KBr for **7b**. 7
Figure S10. 400 MHz ^{1}H-NMR spectrum in d_6-DMSO for **7b**. 7
Figure S11. 400 MHz ^{13}C-NMR spectrum in d_6-DMSO for **7b**. 8
Figure S12. 400 MHz APT spectrum in d_6-DMSO for **7b**. 8
Figure S13. 400 MHz HSQC spectrum in d_6-DMSO for **7b**. 9
Figure S14. 400 MHz HMQC spectrum in d_6-DMSO for **7b**. 9
Figure S15. 400 MHz COSY spectrum in d_6-DMSO for **7b**. 9
Figure S16. High-resolution mass spectrometry -ESI spectrum of **7b**. 10
Figure S17. FT-IR spectrum in KBr for **7c**. 10
Figure S18. 400 MHz ^{1}H-NMR spectrum in d_6-DMSO for **7c**. 11
Figure S19. 400 MHz ^{13}C-NMR spectrum in d_6-DMSO for **7c**. 11
Figure S20. 400 MHz APT spectrum in d_6-DMSO for **7c**. 12
Figure S21. 400 MHz HSQC spectrum in d_6-DMSO for **7c**. 12

Figure S22. 400 MHz HMBC spectrum in d_6-DMSO for **7c**. 12
Figure S23. 400 MHz COSY spectrum in d_6-DMSO for **7c**. 13
Figure S24. High-resolution mass spectrometry -ESI spectrum of **7c**. 13
Figure S25. FT-IR spectrum in KBr for **6d**. 14
Figure S26. 400 MHz ^{1}H-NMR spectrum in d_6-DMSO for **6d**. 14
Figure S27. 400 MHz ^{13}C-NMR spectrum in d_6-DMSO for **6d**. 15
Figure S28. 400 MHz APT spectrum in d_6-DMSO for **6d**. 15
Figure S29. 400 MHz HSQC spectrum in d_6-DMSO for **6d**. 15
Figure S30. 400 MHz HMQC spectrum in d_6-DMSO for **6d**. 16
Figure S31. 400 MHz COSY spectrum in d_6-DMSO for **6d**. 16
Figure S32. High-resolution mass spectrometry -ESI spectrum of **6d**. 16
Figure S33. FT-IR spectrum in KBr for **6e**. 17
Figure S34. 400 MHz ^{1}H-NMR spectrum in d_6-DMSO for **6e**. 17
Figure S35. 400 MHz ^{13}C-NMR spectrum in d_6-DMSO for **6e**. 18
Figure S36. 400 MHz APT spectrum in d_6-DMSO for **6e**. 18
Figure S37. 400 MHz, HSQC spectrum in d_6-DMSO for **6e**. 18
Figure S38. 400 MHz, HMQC spectrum in d_6-DMSO for **6e**. 19
Figure S39. 400 MHz, COSY spectrum in d_6-DMSO for **6e**. 19
Figure S40. High-resolution mass spectrometry -ESI spectrum of **6e**. 19

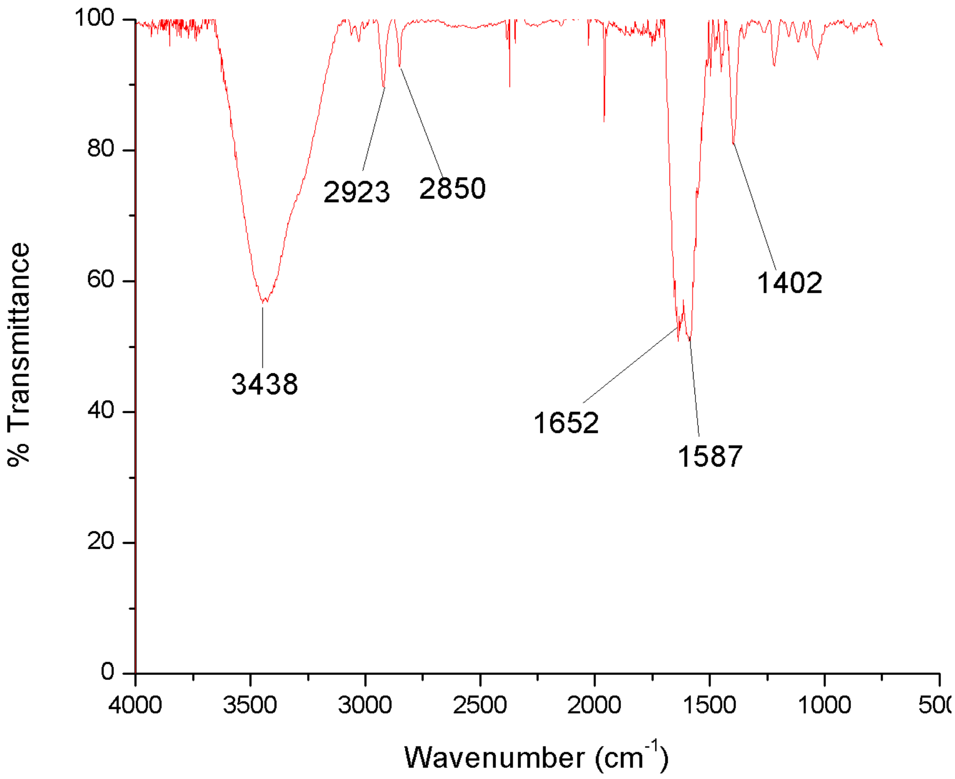

Figure S1. FT-IR spectrum in KBr for **7a**.

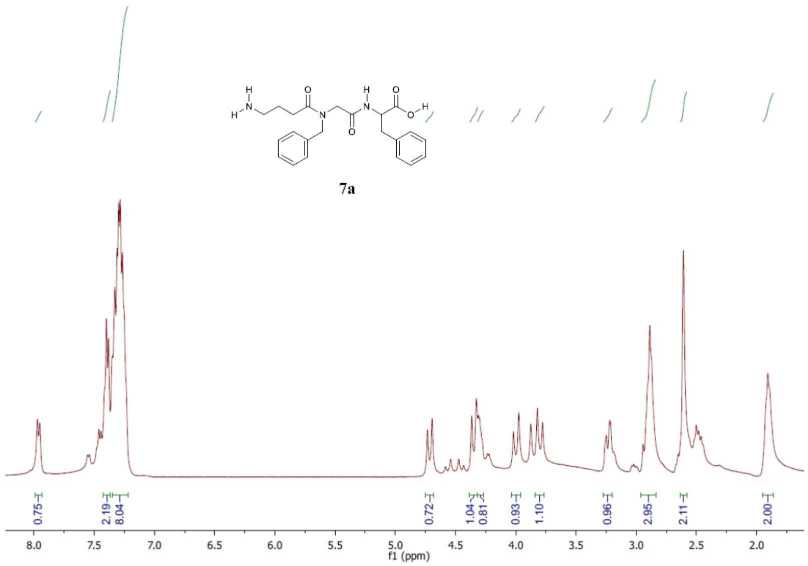

Figure S2. 400 MHz ^{1}H-NMR spectrum in d_6-DMSO for **7a**.

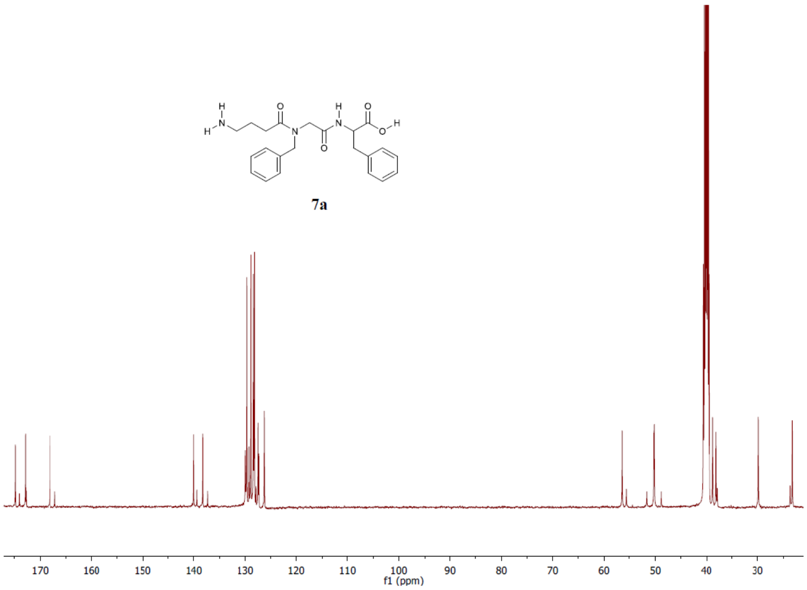

Figure S3. 400 MHz ^{13}C-NMR spectrum in d_6-DMSO for **7a**.

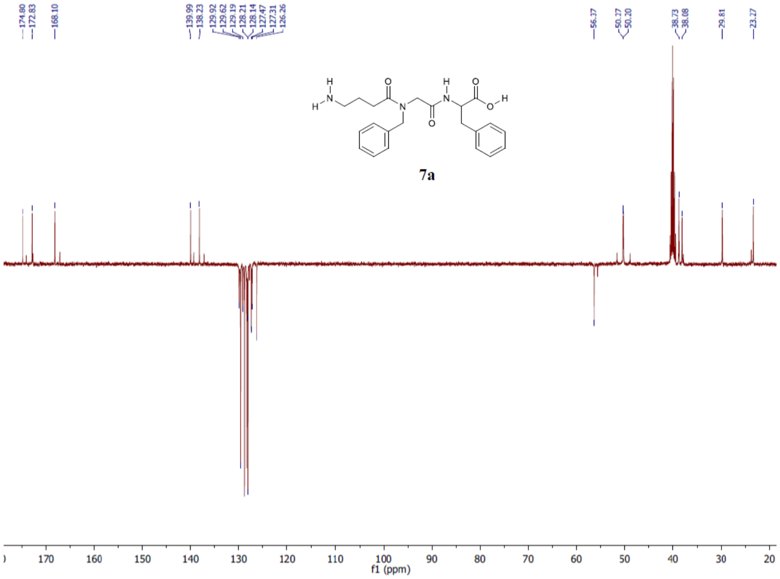

Figure S4. 400 MHz APT spectrum in d_6-DMSO for **7a**.

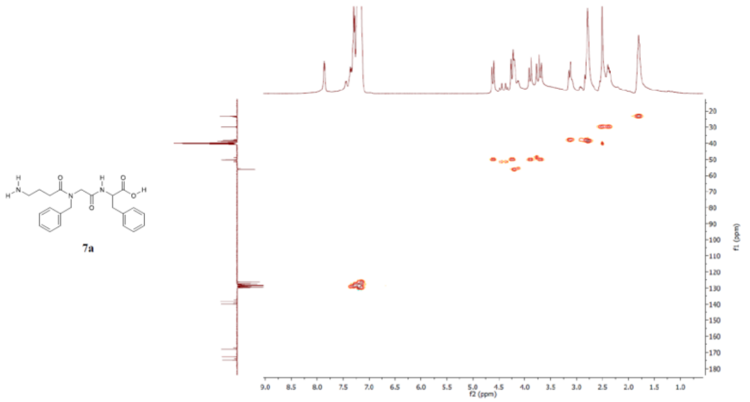

Figure S5. 400 MHz HSQC spectrum in d_6-DMSO for **7a**.

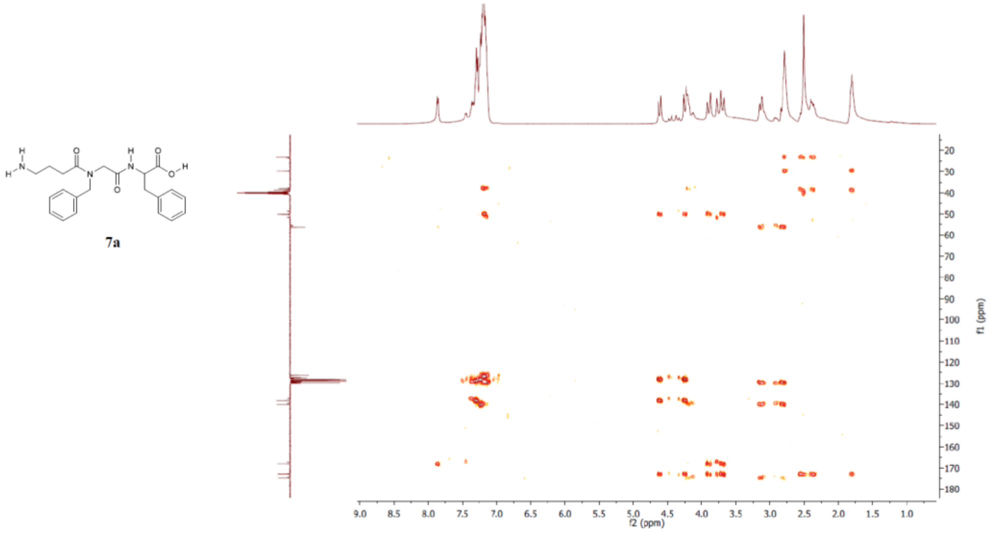

Figure S6. 400 MHz HMBC spectrum in d_6-DMSO for **7a**.

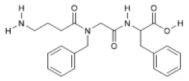

7a

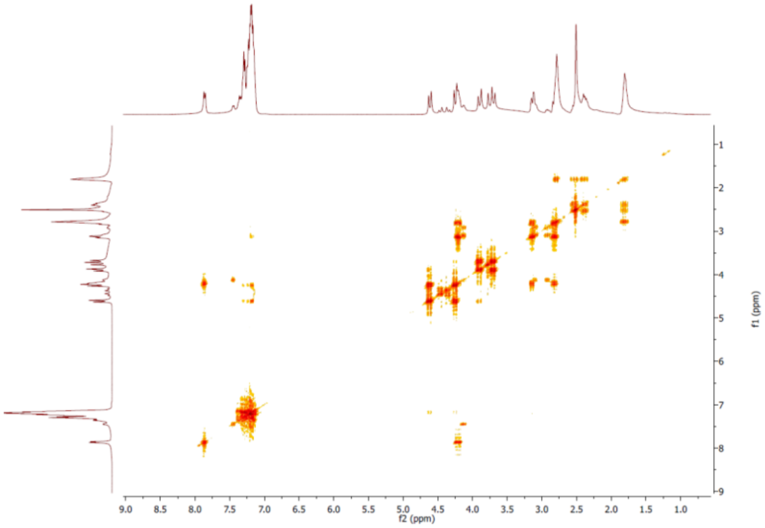

Figure S7. 400 MHz COSY spectrum in d$_6$-DMSO for **7a**.

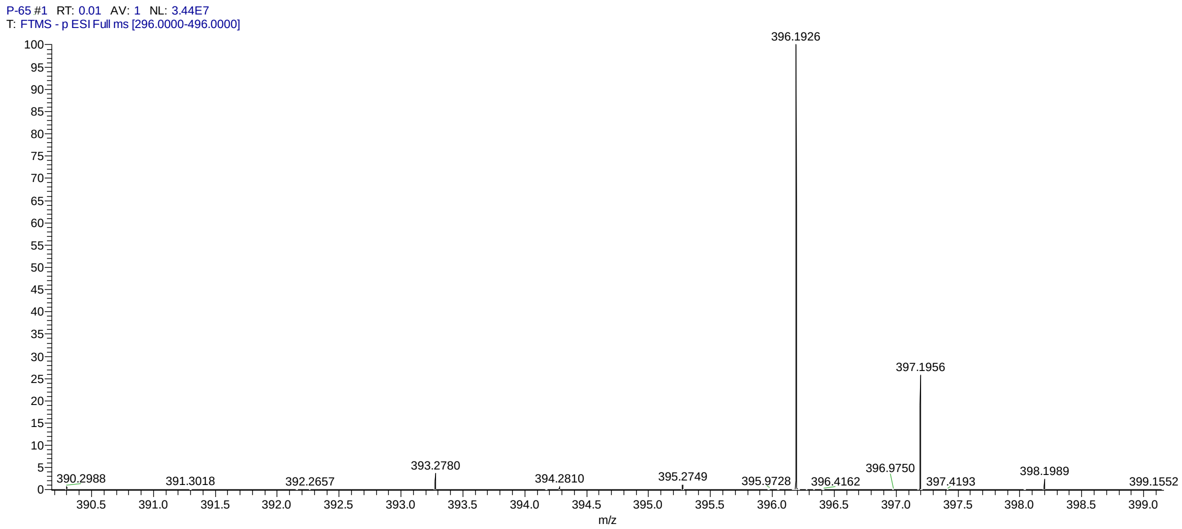

Figure S8. High-resolution mass spectrometry -ESI spectrum of **7a**.

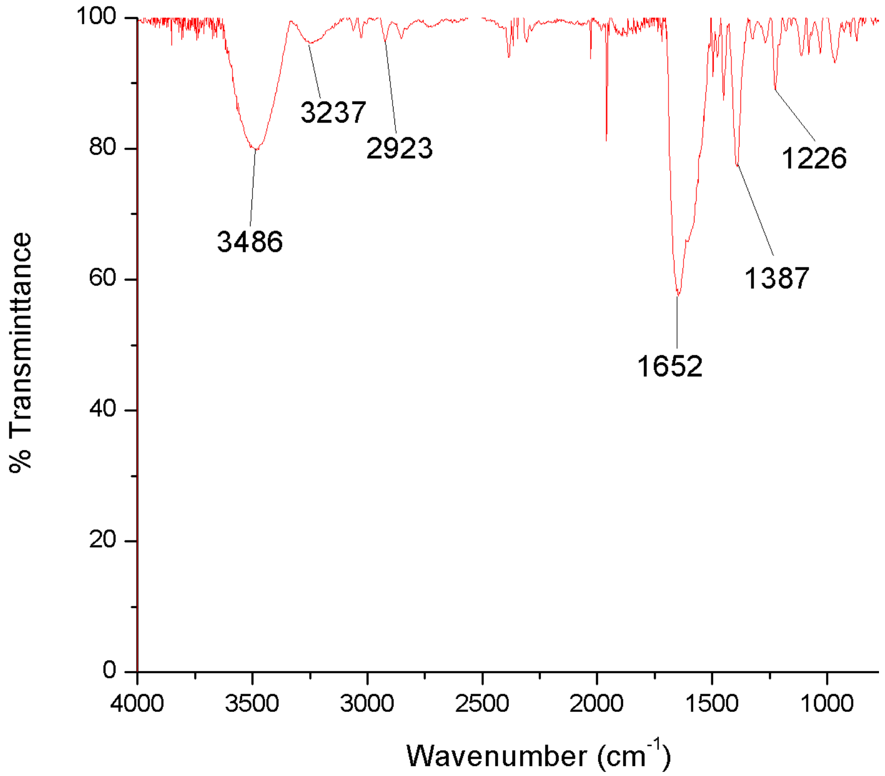

Figure S9. FT-IR spectrum in KBr for **7b**.

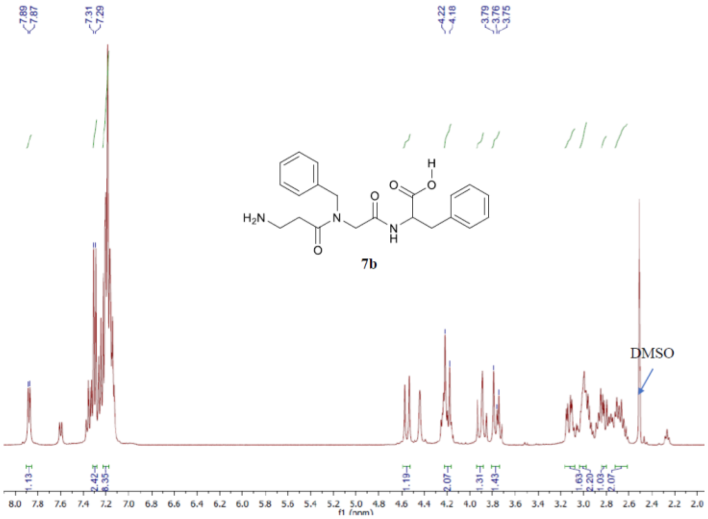

Figure S10. 400 MHz ^{1}H-NMR spectrum in d_6-DMSO for **7b**.

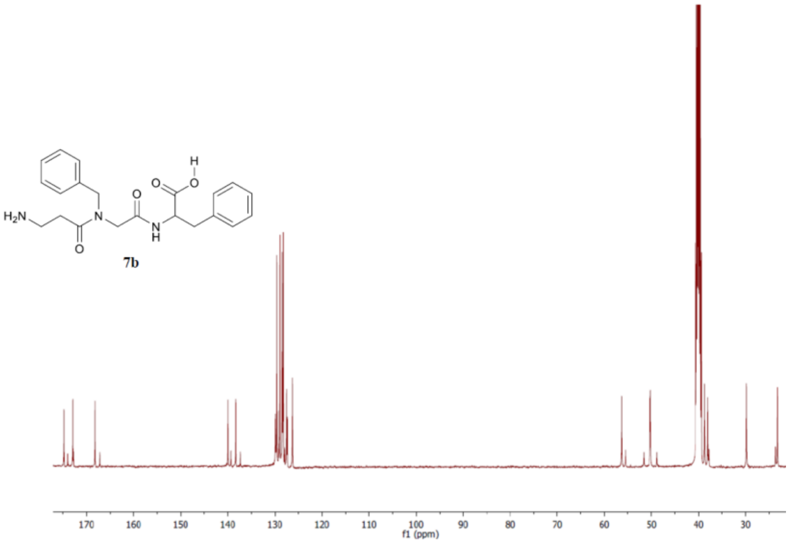

Figure S11. 400 MHz ^{13}C-NMR spectrum in d_6-DMSO for **7b**.

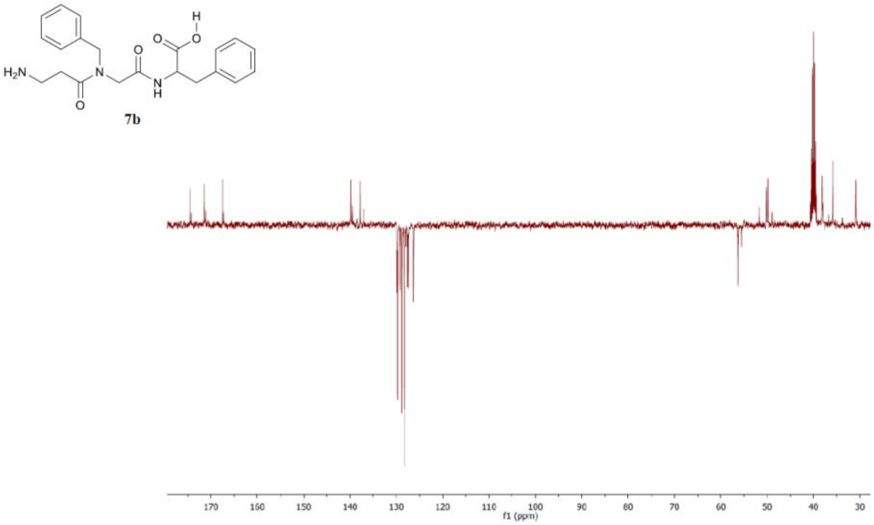

Figure S12. 400 MHz APT spectrum in d_6-DMSO for **7b**.

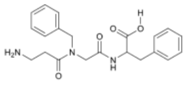

7b

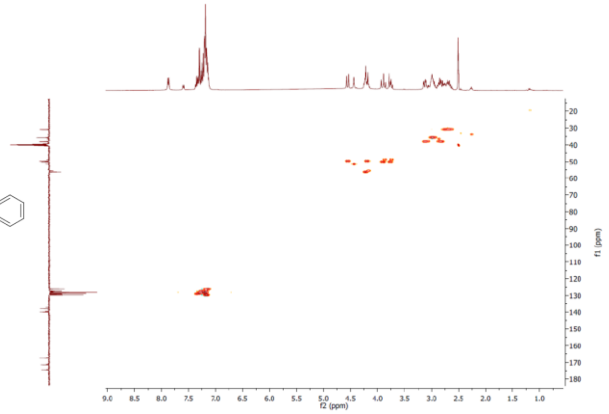

Figure S13. 400 MHz HSQC spectrum in d6-DMSO for **7b**.

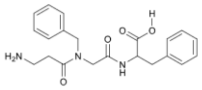

7b

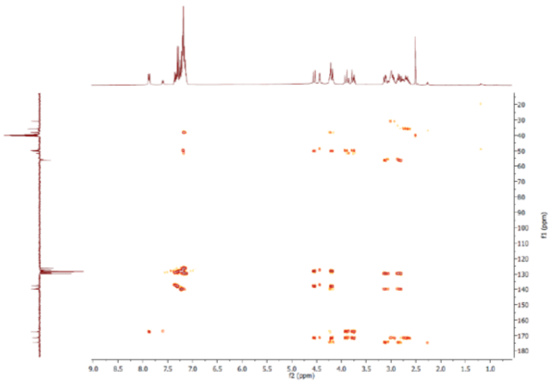

Figure S14. 400 MHz HMQC spectrum in d6-DMSO for **7b**.

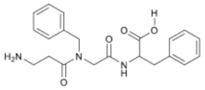

7b

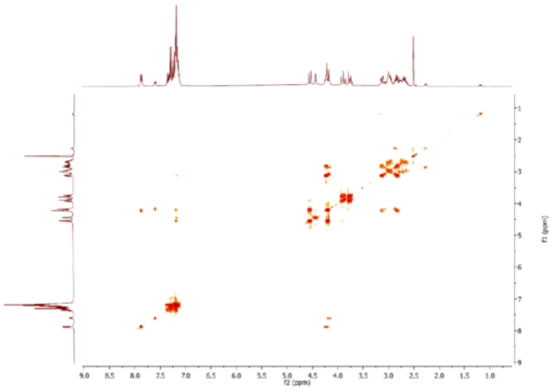

Figure S15. 400 MHz COSY spectrum in d6-DMSO for **7b**.

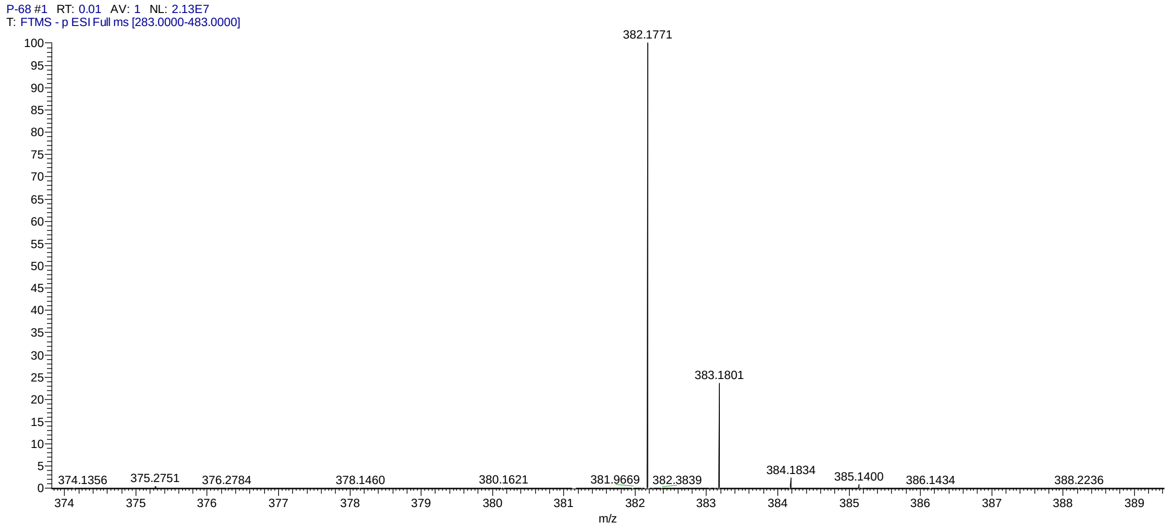

Figure S16. High-resolution mass spectrometry -ESI spectrum of **7b**.

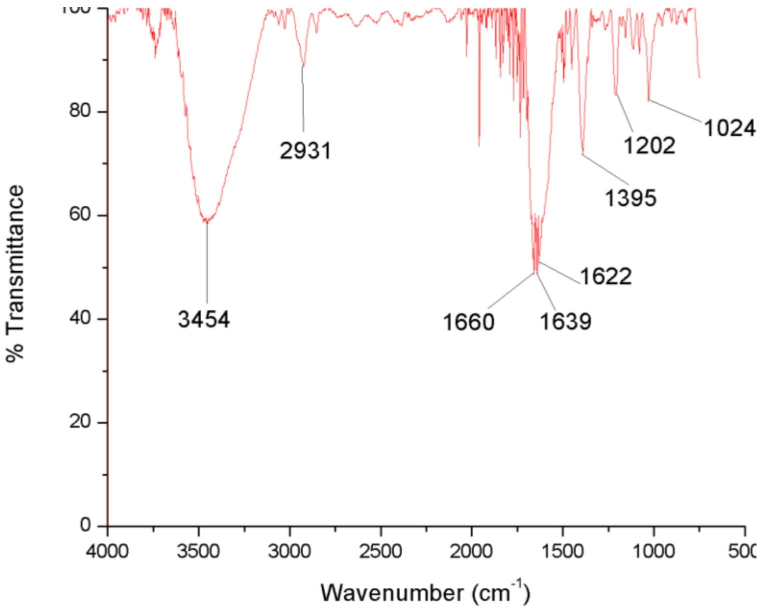

Figure S17. FT-IR spectrum in KBr for **7c**.

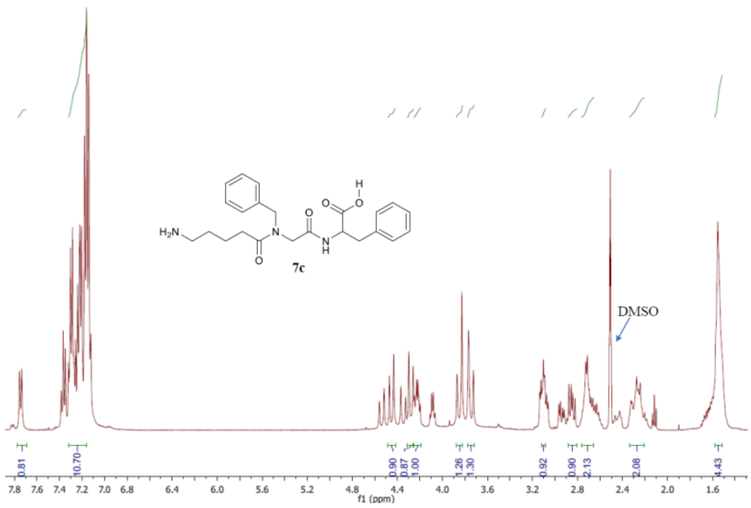

Figure S18. 400 MHz ^{1}H-NMR spectrum in d_6-DMSO for **7c**.

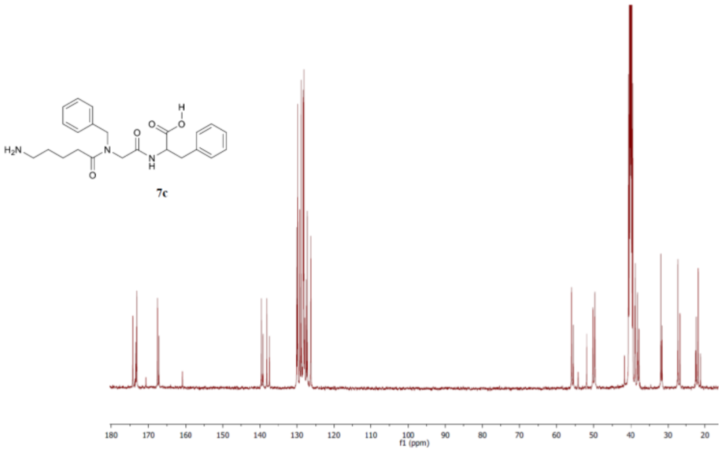

Figure S19. 400 MHz ^{13}C-NMR spectrum in d_6-DMSO for **7c**.

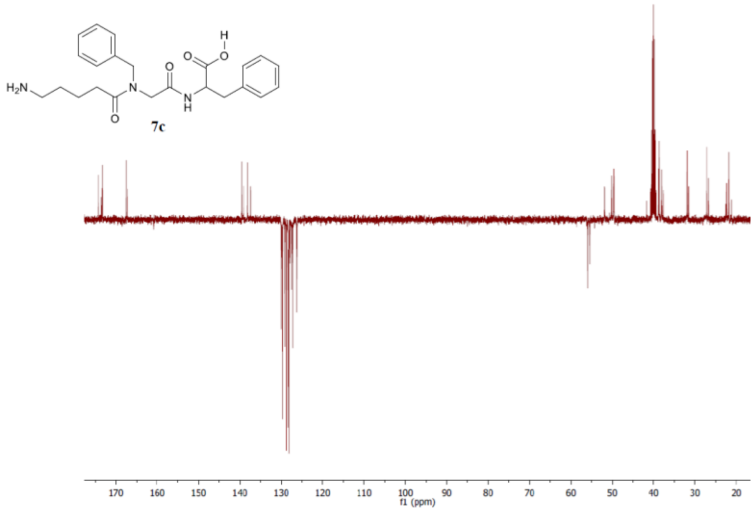

Figure S20. 400 MHz APT spectrum in d_6-DMSO for **7c**.

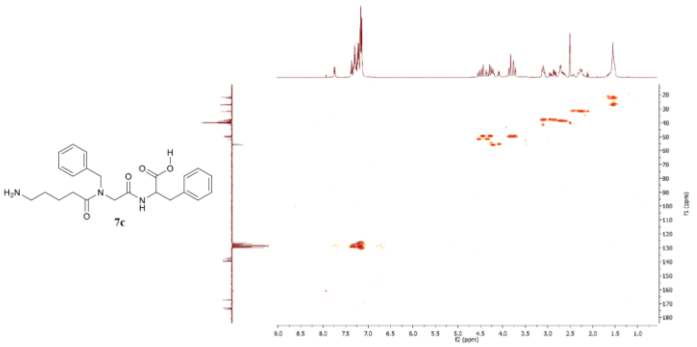

Figure S21. 400 MHz HSQC spectrum in d_6-DMSO for **7c**.

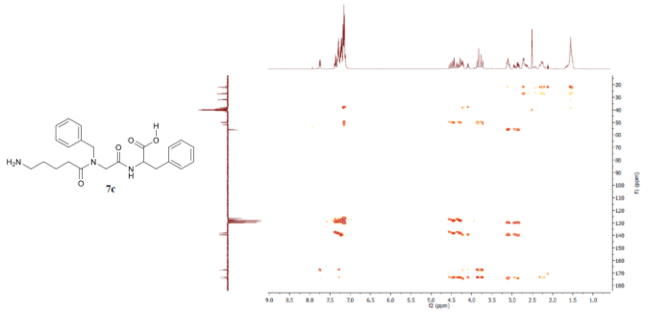

Figure S22. 400 MHz HMBC spectrum in d_6-DMSO for **7c**.

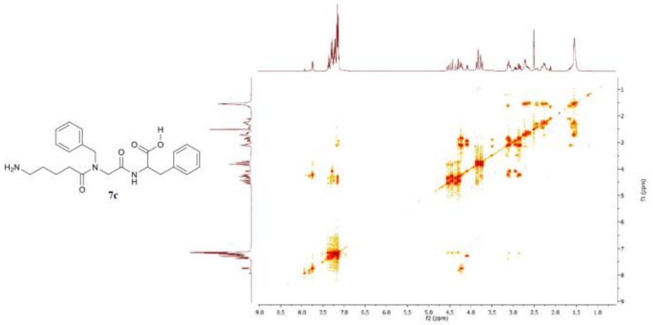

Figure S23. 400 MHz COSY spectrum in d6-DMSO for **7c**.

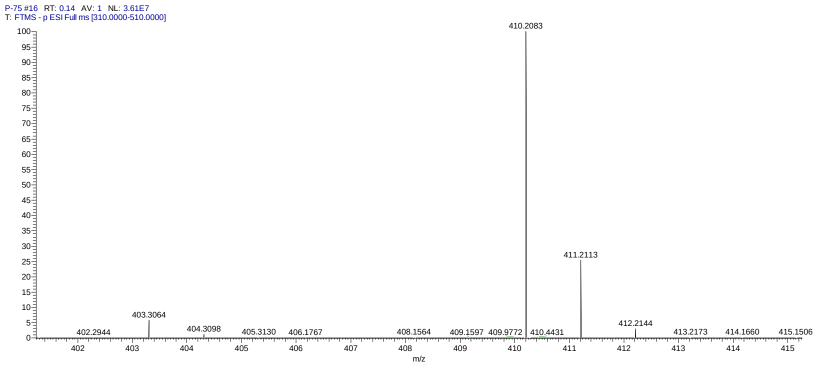

Figure S24. High-resolution mass spectrometry -ESI spectrum of **7c**.

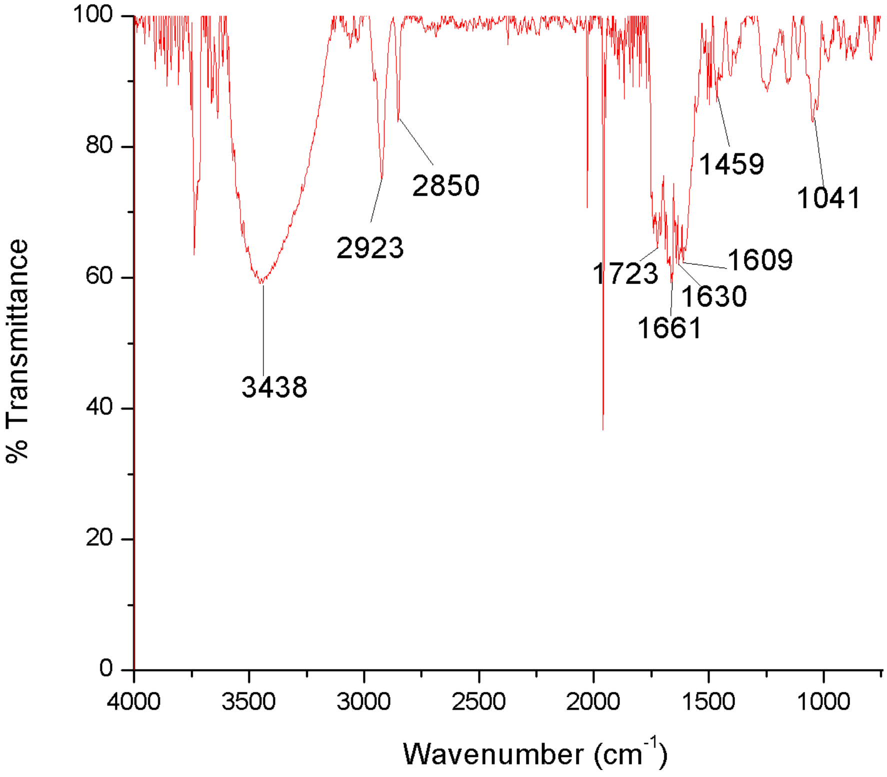

Figure S25. FT-IR spectrum in KBr for **6d**.

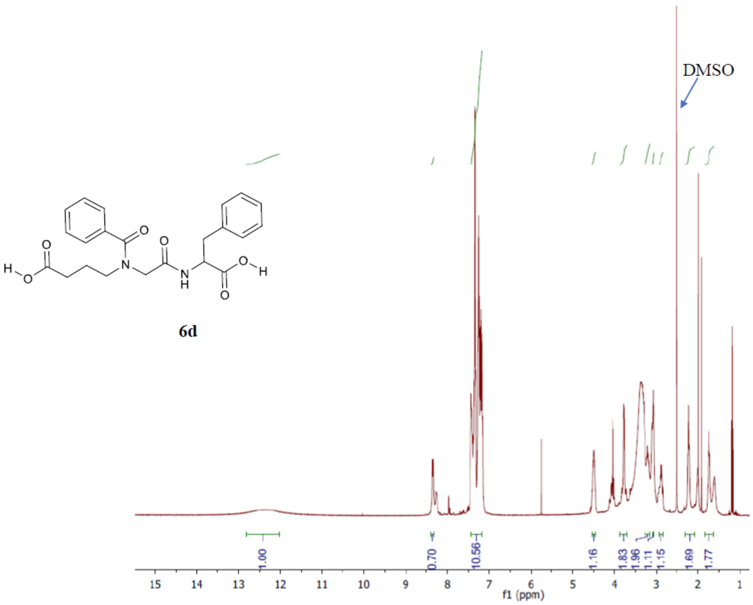

Figure S26. 400 MHz ^{1}H-NMR spectrum in d_6-DMSO for **6d**.

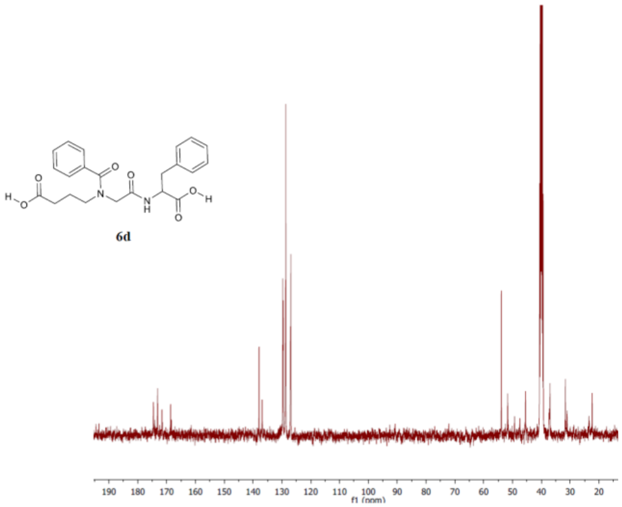

Figure S27. 400 MHz ^{13}C-NMR spectrum in d_6-DMSO for **6d**.

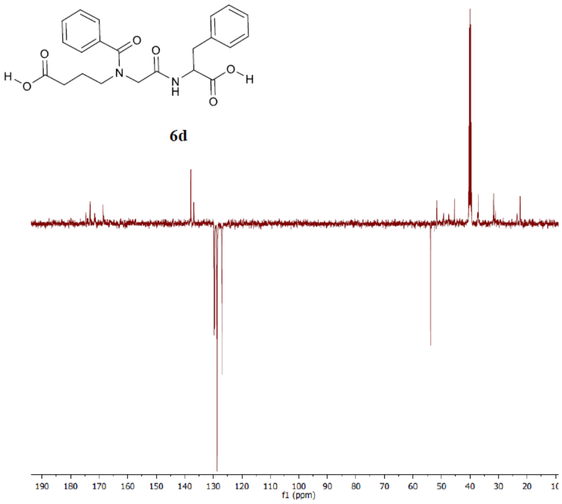

Figure S28. 400 MHz APT spectrum in d_6-DMSO for **6d**.

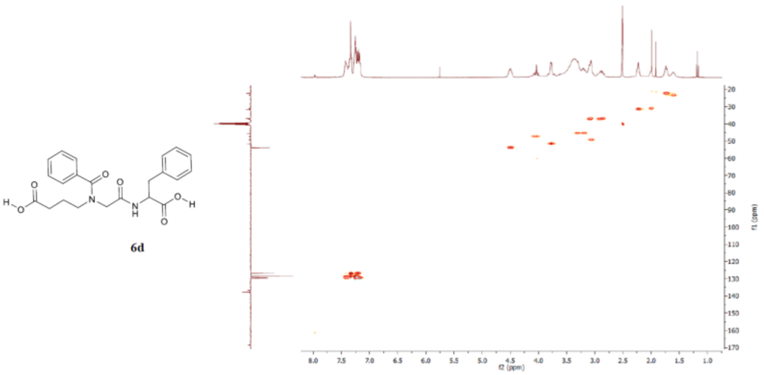

Figure S29. 400 MHz HSQC spectrum in d_6-DMSO for **6d**.

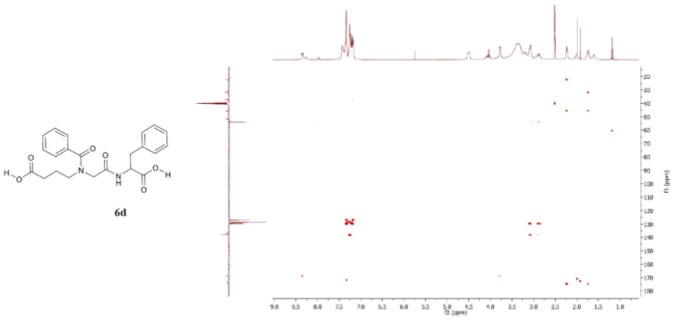

Figure S30. 400 MHz HMQC spectrum in d_6-DMSO for **6d**.

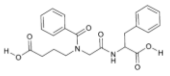

6d

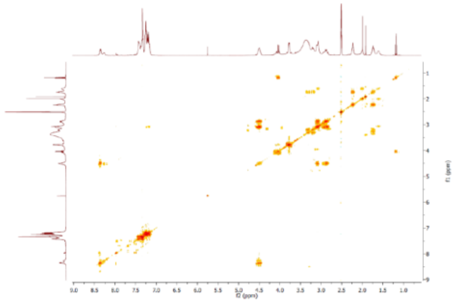

Figure S31. 400 MHz COSY spectrum in d6-DMSO for **6d**.

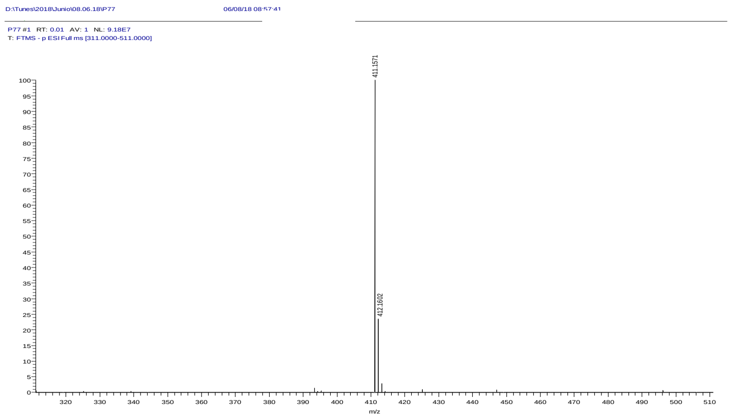

Figure S32. High-resolution mass spectrometry -ESI spectrum of **6d**.

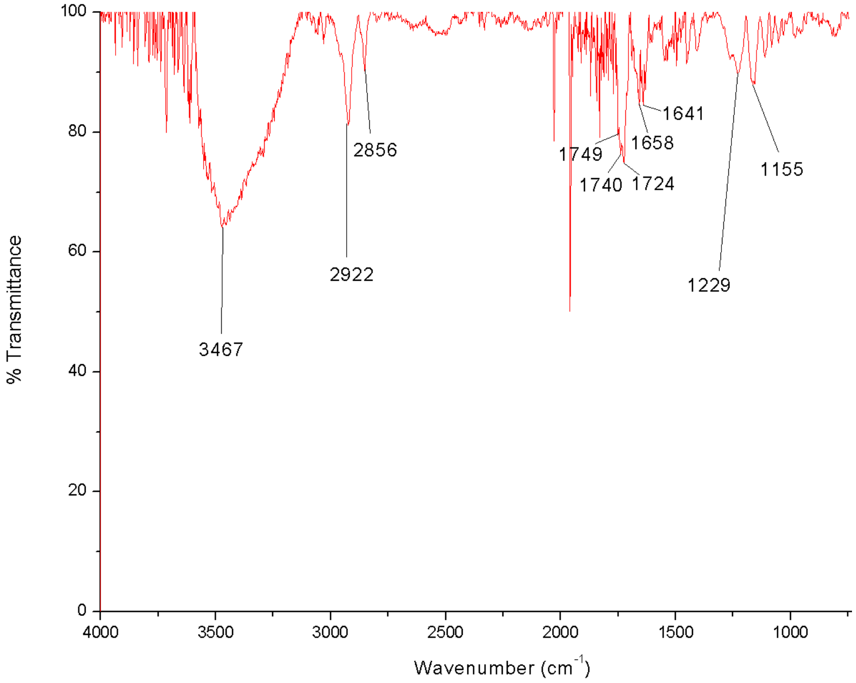

Figure S33. FT-IR spectrum in KBr for **6e**.

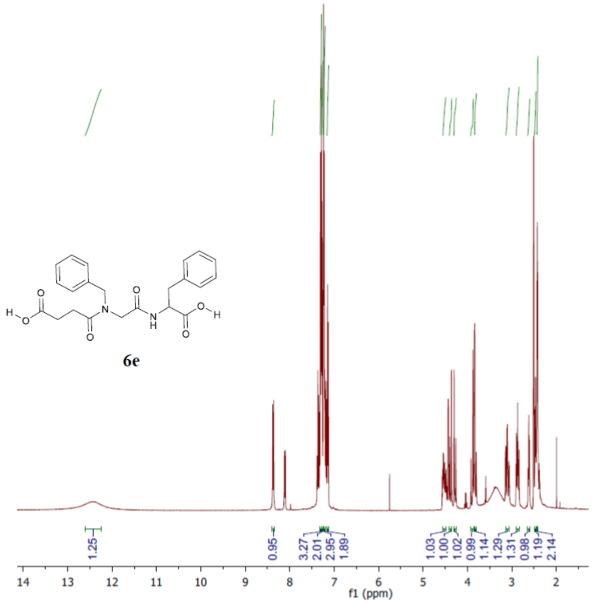

Figure S34. 400 MHz ^{1}H-NMR spectrum in d_6-DMSO for **6e**.

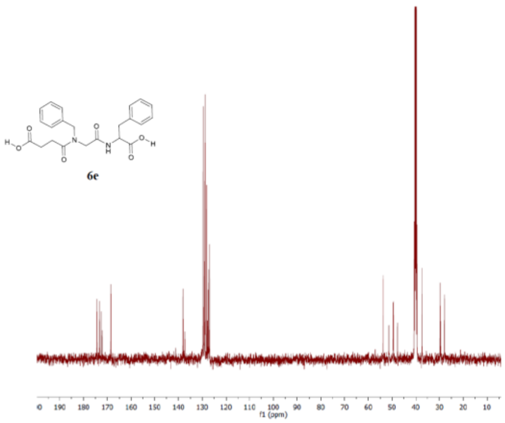

Figure S35. 400 MHz ^{13}C-NMR spectrum in d_6-DMSO for **6e**.

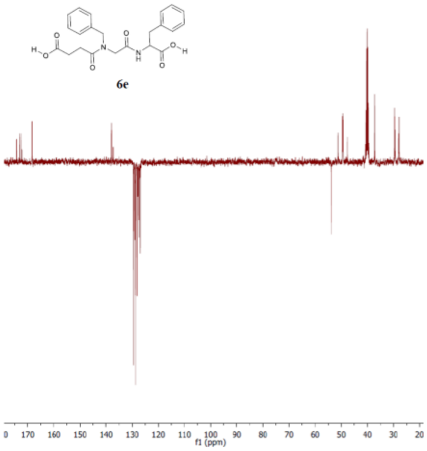

Figure S36. 400 MHz APT spectrum in d_6-DMSO for **6e**.

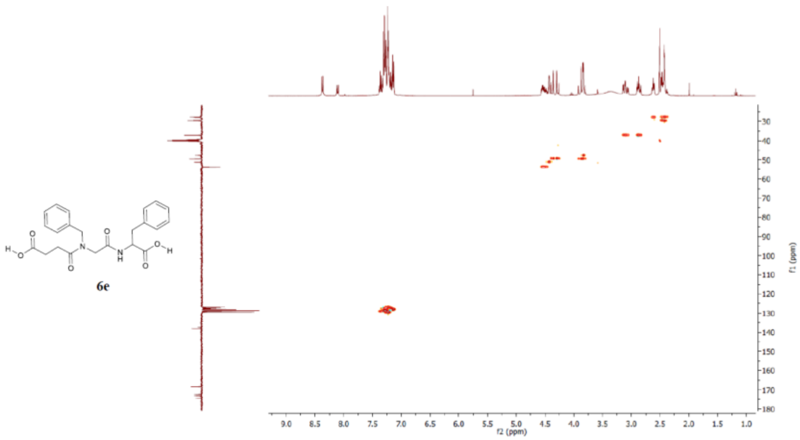

Figure S37. 400 MHz, HSQC spectrum in d_6-DMSO for **6e**.

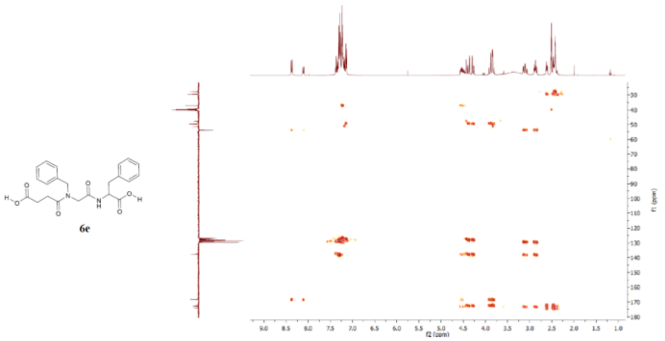

Figure S38. 400 MHz, HMQC spectrum in d6-DMSO for **6e**.

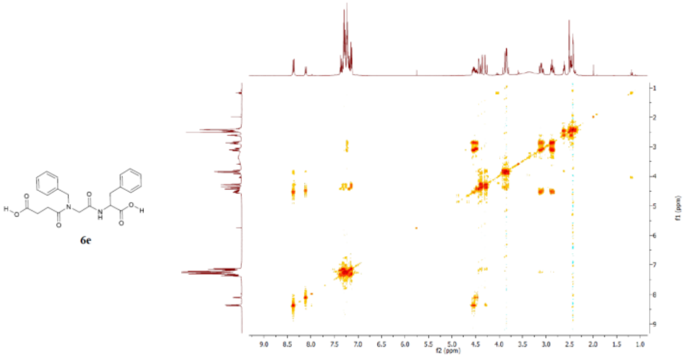

Figure S39. 400 MHz, COSY spectrum in d6-DMSO for **6e**.

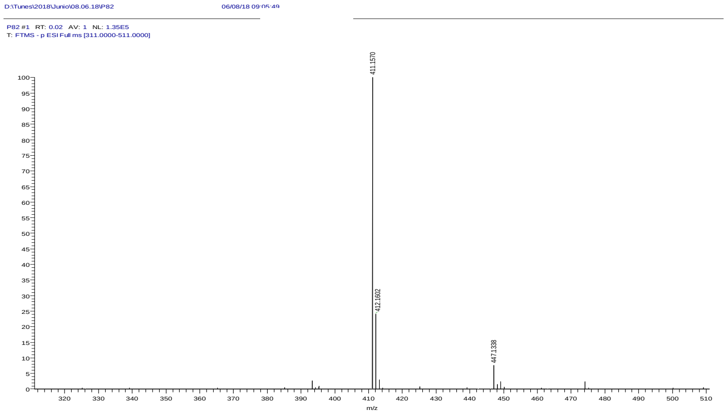

Figure S40. High-resolution mass spectrometry -ESI spectrum of **6e**.
